# Invasive FoxM1 phosphorylated by PLK1 induces the polarization of tumor-associated macrophages to promote immune escape and metastasis, amplified by IFITM1

**DOI:** 10.1186/s13046-023-02872-1

**Published:** 2023-11-16

**Authors:** Rong Xu, Young-Joo Lee, Chang-Hyeon Kim, Ga-Hong Min, Yeo-Bin Kim, Jung-Won Park, Dae-Hoon Kim, Jung-Hyun Kim, Hyungshin Yim

**Affiliations:** 1 Department of Pharmacy, College of Pharmacy, Institute of Pharmaceutical Science and Technology, Hanyang University, Ansan, Gyeonggi-Do 15588 Republic of Korea; 2 Division of Intractable Diseases Research, Department of Chronic Diseases Convergence Research, Korea National Institute of Health, Cheongju, Chungcheongbuk-Do 28160 Republic of Korea

**Keywords:** FoxM1, Phosphorylation, PLK1, Invasiveness, Tumor-associated macrophages

## Abstract

**Background:**

Understanding the mechanism behind immune cell plasticity in cancer metastasis is crucial for identifying key regulators. Previously we found that mitotic factors regulate epithelial-mesenchymal transition, but how these factors convert to metastatic players in the tumor microenvironment (TME) is not fully understood.

**Methods:**

The clinical importance of mitotic factors was analyzed by heatmap analysis, a KM plot, and immunohistochemistry in lung adenocarcinoma (LUAD) patients. Immunoprecipitation, LC–MS/MS, kinase assay, and site-directed mutagenesis were performed for the interaction and phosphorylation. A tail-vein injection mouse model, Transwell-based 3D culture, microarray analysis, coculture with monocytes, and chromatin immunoprecipitation assays were used to elucidate the function of phosphorylated FoxM1 in metastasis of TME.

**Results:**

The phosphorylated FoxM1 at Ser25 by PLK1 acquires the reprogramming ability to stimulate the invasive traits in cancer and influence immune cell plasticity. This invasive form of *p*-FoxM1 upregulates the expression of IL1A/1B, VEGFA, and IL6 by direct activation, recruiting monocytes and promoting the polarization of M2d-like tumor-associated macrophages (TAMs). Upregulation of PD-L1 in LUAD having phosphomimetic FoxM1 facilitates immune evasion. In invasive LUAD with phosphomimetic FoxM1, *IFITM1* is the most highly expressed through the activation of the STING-TBK1-IRF3 signaling, which enhances FoxM1-mediated signaling. Clinically, higher expression of *FOXM1*, *PLK1*, and *IFITM1* is inversely correlated with the survival rate of advanced LUAD patients, providing a promising therapeutic strategy for the treatment of LUAD.

**Conclusion:**

FoxM1-based therapy would be a potential therapeutic strategy for LUAD to reduce TAM polarization, immune escape, and metastasis, since FoxM1 functions as a genetic reprogramming factor reinforcing LUAD malignancy in the TME.

**Supplementary Information:**

The online version contains supplementary material available at 10.1186/s13046-023-02872-1.

## Background

The predominant cause of the high mortality in cancer patients is metastasis, responsible for up to 90% of cancer deaths [1]. Understanding the regulatory mechanisms of metastasis is important for developing therapeutic strategies for cancer [2, 3]. During metastasis, cancer cells undergo an epithelial-mesenchymal transition (EMT) driven by genetic reprogramming and posttranslational modification to allow invasion and migration, which require crosstalk with the local tumor microenvironment (TME) [3–5]. Communications between cancer cells and immune cells, such as macrophages and T lymphocytes, of the TME play critical roles in malignancy, metastasis, and immune escape of cancer as well as polarization of immune cells by releasing cytokines and chemokines [6, 7]. Exploring the mechanism of the plasticity of immune cells triggered by cancer cells through crosstalk is important for discovering key regulators to manage cancer metastasis.

Genetic reprogramming is critical to acquire the mesenchymal characteristics during metastasis that affect the TME through crosstalk between cells. Evidence suggests that mitotic transcriptional factor FoxM1 functions as the putative EMT regulator by activation of EMT transcriptional factors including SNAI1 and SNAI2 [8–12]. FoxM1 upregulation in the majority of carcinomas [13–18] is involved in tumor malignancy [15, 17, 19] and the poor clinical prognosis by stimulating metastasis [20, 21]. In several carcinomas, loss of FoxM1 leads to reduction of cancer invasion and migration by blockage of the EMT [12, 22–24]. FoxM1 regulates the expression of mitotic factors including cyclin B1 and PLK1 [25–28]. Reciprocally, its activity is regulated by PLK1-mediated phosphorylation in mitosis [28]. Recent evidence showed that mitotic factors induce EMT [29–31], but it is incompletely understood how these factors are converted into metastatic players in the TME.

Posttranslational modification is a crucial regulatory mechanism for tumorigenesis and the EMT [31, 32]. A main mitotic kinase PLK1 is a therapeutic target for most carcinomas due to its driving effects in metastasis and tumorigenesis [31, 33–35]. Many studies have supported the involvement of PLK1 signaling in the EMT and metastasis of esophageal squamous cell, gastric, prostate, and non-small cell lung cancer (NSCLC) [29, 36–38]. Active PLK1 directly drives metastasis through the activation of transforming growth factor (TGF) β signaling [29]. Cytoskeletal vimentin phosphorylated by PLK1 facilitates immune escape by recruiting Smad2/3 to PD-L1 promoter for its expression [30]. However, the function of tumorigenic protein kinase, even PLK1, in crosstalk with cells of the TME is not understood.

To explore this, we investigated potential pathway crosstalk nodes and factors in EMT. Here, we demonstrate that p-FoxM1^S25^ functions in genetic reprogramming from primary to metastatic cancer by activating TAMs polarization, immune escape, and metastasis as a direct transcriptional factor reinforcing LUAD malignancy in the TME.

## Materials and methods

### Materials

A549, NCI-H1299, NCI-H358, NCI-H460, HCC827, and THP-1 cells were purchased from KCLB (KCLB; Seoul, Korea). Minimum essential medium Eagle (MEM), RPMI 1640 medium, Dulbecco's modified Eagle's medium (DMEM), fetal bovine serum (FBS), penicillin, and streptomycin were purchased from Corning Life Sciences (Tewksbury, MA, USA). Trametinib, ruxolitinib, and fludarabine were purchased from Selleck Chemical (Houston, TX, USA), and BI605906 was purchased from Universal Biologicals (London, UK). TGF-β and all other chemical reagents were purchased from Sigma-Aldrich (St. Louis, MO, USA).

### Cell culture and treatment

A549, NCI-H358, NCI-H460, NCI-H1299, and HCC827 cells were cultured in RPMI 1640 medium. BEAS-2B human bronchial epithelial cells (ATCC #CRL-9609) provided from Prof. Moon (Sejong University, Korea) were cultured in DMEM [39]. The cells were supplemented with 10% FBS, 100 units/mL penicillin, and 100 units/mL streptomycin in a humidified 5% CO_2_ incubator at 37 ℃. For TGF-β treatment, cells were seeded at 5 × 10^4^ cells/mL and treated with 5 ng/mL TGF-β for 48 h at 24 h later. Human macrophage cells were differentiated from the human embryonic stem cell line H9 obtained from WiCell Research Institute (Madison, WI, USA) following the previously reported method [40]. Human macrophage cells were maintained in RPMI 1640 containing 100 ng/ml human macrophage colony-stimulating factor.

### Immunoblot analysis

After treatment with TGF-β for 48 h, cells were lysed with lysis buffer [0.5% Triton X-100, 20 mM Tris, pH 7.5, 2 mM MgCl_2_, 1 mM dithiothreitol (DTT), 1 mM egtazic acid (EGTA), 50 mM β-glycerophosphate, 25 mM NaF, 1 mM Na_3_VO_4_, 100 mg/mL phenylmethylsulfonyl fluoride, and protease inhibitor cocktail] (Roche; Indianapolis, IN, USA). Proteins were resolved by SDS-PAGE and subjected to immunoblot analysis with specific antibodies as follows (Table S1): FoxM1 (Santa Cruz Biotechnology, sc500; Santa Cruz, CA, USA); PLK1 (Millipore, 05-844; Billerica, MA, USA); p-PLK1-T210 (Cell Signaling, 5472S; Danvers, MA, USA); vimentin (Santa Cruz Biotechnology, sc7557); E-cadherin (Cell Signaling, 4065); N-cadherin (Sigma-Aldrich, C3865); SNAI1 (Santa Cruz Biotechnology, sc271977); SNAI2 (Cell Signaling, 9585); GAPDH (Sigma-Aldrich, G8795); p-Smad2 S465/S467 (Cell Signaling, 18338); Smad2/3 (Cell Signaling, 8685); β-actin (Sigma-Aldrich, A5441), TCTP (Santa Cruz Biotechnology, sc133131); p-TCTP (Cell Signaling, 5251s); RFP (Life Technologies, R10367; Carlsbad, CA, USA); PD-L1 (Cell Signaling, 13684); p-Erk1/2 (Cell Signaling, 4370p); Erk1/2 (Cell Signaling, 4695p); c-Fos (Santa Cruz Biotechnology, sc52); c-Jun (Santa Cruz Biotechnology, sc74543); IFITM1 (ProteinTech, 60074-1-lg; Rosemont, IL, USA); IL1A (Santa Cruz Biotechnology, sc12741); STAT1 (Santa Cruz Biotechnology, sc464); NF-kB (Santa Cruz Biotechnology, sc71677); Histone H1 (Santa Cruz Biotechnology, sc8030); STING (Santa Cruz Biotechnology, sc518172); TBK1 (Santa Cruz Biotechnology, sc52957); p-TBK1 (Cell Signaling, 5483t); and IRF3 (Santa Cruz Biotechnology, sc33641). Immune complexes were detected with an Odyssey infrared imaging system, (LI-COR Biosciences; Lincoln, NE, USA). Intensity values were determined with LI-COR Odyssey software.

### Quantitative real-time polymerase chain reaction (qRT-PCR)

Total RNA was extracted from cells and tissues using TRIzol™ reagent (Thermo Fisher Scientific) according to the manufacturers’ direction. cDNA was synthesized using a First Strand cDNA Synthesis Kit (Thermo Fisher Scientific, Waltham, MA, USA) and then mixed with SYBR Green Master Mix (Bio-Rad Laboratories, Hercules, CA, USA) and gene-specific primers to perform qRT-PCR using a CFX96 Real-Time PCR system (Bio-Rad Laboratories). The primer sequences used are shown in Table S2. The results were analyzed using the ∆∆Ct method with GAPDH, a housekeeping gene, as a control.

### Immunoprecipitation assay

Cells lysates were incubated with normal IgG (Santa Cruz Biotechnology), anti-PLK1 (Millipore, 05–844), anti-c-Myc polyclonal (Sigma-Aldrich, c3956), or anti-FoxM1 (Santa Cruz Biotechnology, sc-500) antibodies for 16 h at 4 °C with end-over-end mixing, followed by incubation with protein A agarose (Santa Cruz Biotechnology) for 3 h at 4 °C. Immunoprecipitates were separated from supernatants by centrifugation and washed four times with lysis buffer. Proteins were resolved by SDS-PAGE and analyzed by immunoblot with anti-FoxM1 (Santa Cruz Biotechnology, sc-500); anti-PLK1 (Millipore, 05–844); anti-c-Myc monoclonal (Santa Cruz Biotechnology, sc-40); β-actin (Sigma-Aldrich, A5441); or anti-GAPDH (Sigma-Aldrich, G8795) antibodies.

### Kinase assay

A Plk1 kinase assay was performed with the constitutively active form of Plk1 (T210D), [γ-^32^P] ATP, and glutathione-S-transferase (GST)-tagged FoxM1 as a substrate. For purification of GST-tagged FoxM1, FoxM1 plasmids were sub-cloned into pGEX-4 T-1 vector and expressed in *Escherichia coli* BL21 strain. The proteins were purified with glutathione agarose (50% v/v) and eluted with buffer containing 10 mM reduced glutathione. Plk1 kinase and substrates were incubated in kinase buffer (50 mM Tris–HCl, pH 7.5, 10 mM MgCl_2_, 5 mM DTT, 2 mM EGTA, 0.5 mM Na_3_VO_4_, and 20 mMβ-glycerophosphate) containing 25 μM ATP and 5 μCi [γ-^32^P] ATP for 30 min at 30 °C. GST-tagged TCTP protein was used as a positive control. The reacted samples were suspended in SDS loading buffer, resolved by SDS-PAGE, and detected by autoradiography.

### Enrichment of phosphorylated FoxM1 protein

A Plk1 kinase assay was performed using active Plk1 and GST-tagged FoxM1 in reaction buffer containing 25μM ATP at 30 °C for 30 min. The phosphorylated GST-FoxM1 proteins were resolved by SDS-PAGE, and the bands from SDS-PAGE were in-gel digested with trypsin. After an overnight incubation in 25 mM ammonium bicarbonate buffer at pH 7.8 and 37 ℃, the tryptic peptides were extracted with 5 μL of 0.5% trifluoroacetic acid containing 50% (v/v) acetonitrile (ACN) for 40 min with mild sonication. The extracted solution was concentrated using a centrifugal vacuum concentrator. Prior to mass spectrometric analysis, the peptide solution was subjected to a desalting process using a reverse-phase column. The bound peptides were eluted with 5 μL of 70% ACN with 5% (v/v) formic acid.

### LC–MS/MS analysis

After in-gel digestion and extraction of the phosphorylated peptides, nano LC–MS/MS analysis was performed with an Easy n-LC (Thermo Fisher Scientific) and an LTQ Orbitrap XL mass spectrometer (Thermo Fisher Scientific) equipped with a nano-electrospray source. Samples were separated on a C18 nanobore column (150 mm × 0.1 mm, 3 μm pore size, Agilent). The mobile phase A for LC separation was 0.1% formic acid and 3% acetonitrile in deionized water and the mobile phase B was 0.1% formic acid in acetonitrile. The chromatography gradient was designed for a linear increase from 5 to 55% B in 40 min, 52 to 75% B in 4 min, 95% B in 4 min, and 3% B in 6 min. The flow rate was maintained at 1500 mL/min. Mass spectra were acquired using data dependent acquisition with a full mass scan (350–1200 m/z) followed by 10 MS/MS scans. For MS1 full scans, the orbitrap resolution was 15,000 and the AGC was 2 × 10^5^. For MS/MS in the LTQ, the AGC was 1 × 10^4^. The individual spectra from MS/MS were processed using SEQUEST software (Thermo Fisher Scientific), and the generated peak lists were used to query using the MASCOT program (Matrix Science Ltd., London, UK). The tolerance of the peptide mass was set to 10 ppm. MS/MS ion mass tolerance was 0.8 Da, allowance of missed cleavage was 2, and charge states (+ 2 and + 3) were considered for data analysis. We used only significant hits as defined by MASCOT probability analysis.

### Generation of phosphomimetic and non-phosphomimetic mutants of FoxM1

Site-directed mutagenesis was carried out using a QuikChange II multi-site-directed mutagenesis kit (Agilent, #200,524; Santa Clara, CA, USA) according to the manufacturer's protocol. The mutagenic primer sequences are shown in Table S3.

### Lentivirus-based plasmid preparation, virus production, and infection

For expression of human FoxM1, plasmids for wild-type and mutants of human FoxM1B (gene access no. U74613) were inserted in pLVX-TRE3G (Clontech, Mountain View, CA, USA). Lentivirus was prepared according to the manufacturer’s manual. To generate doxycycline-inducible FoxM1-expressing cell lines, lentivirus from pLVX-TRE3G-eRFP-FoxM1 and pLVX-Tet3G (Clontech) was used, as described previously [29]. The infected cells were selected using 500 µg/mL G418 for 5 days and 2 µg/mL puromycin for 2 days. FoxM1 expression was induced by treatment with 2 µg/mL doxycycline (Fig. S1).

### Lentivirus-based shRNA preparation

For loss of function experiments, lentivirus-based shRNA targeting human FoxM1B (gene access no. U74613) at positions 187–207 (CATCAGAGGAGGAACCTAAGA) (pLKO-Puro.1-FoxM1#187) or at positions 709–729 (CCTTTTCCTCCATCTCTTGCT) (pLKO-Puro.1-FoxM1#709) and human IFITM1 (gene access no. NM_003641) at positions 373–393 (CCGCCAAGTGCCTGAACATCT) (pLKO-Puro.1-IFITM1#373) or at 413–433 (CCTCATGACCATTGGATTCAT) (pLKO-Puro.1-IFITM1#413) was prepared, and the lentivirus was generated as described previously [30]. The infected cells were selected using 2 µg/mL puromycin for 2 days (Fig. S1).

### Transwell cell migration and invasion assay

Cell migration assays were conducted using 24-well plates with 8-μm pore Transwell chambers (Corning Life Sciences). The lower chamber was filled with culture medium containing 10% FBS. A549 or NCI-H460 cells were suspended at a density of 5 × 10^4^ cells or 1 × 10^5^ cells, respectively, in RPMI medium without FBS and added to the upper chamber. Three days after seeding, the cells on the bottom surface were stained with 0.05% crystal violet dye, and the intensity values were measured using an Odyssey infrared imaging system. For the cell invasion assay, cells were seeded in the upper chamber filled with Matrigel (BD Biosciences; Erembodegem, Aalst, Belgium). Seven days after seeding, the cells on the bottom surface were stained with 0.05% crystal violet dye.

### Experimental lung metastasis assay

Four-week-old male BALB/c nude mice (Orient Bio, Seoul, Korea) were injected with A549 cells stably expressing pLVX-TRE3G-eRFP-Tet3G-tagged mock, wild-type, S25A, or S25E FoxM1 (2 × 10^6^ cells in 100 μL phosphate-buffered saline (PBS)) via the tail vein. The mice received 1 mg/mL of doxycycline in their drinking water to induce TRE3G-FoxM1 overexpression. Fifteen weeks after injection, all mice were sacrificed, and their lungs were separated and fixed in 4% paraformaldehyde for H&E, Ki67, CD68, and CD163 tissue staining. All animal experiments were approved and managed by the guidelines of the Institutional Animal Care and Use Committee, Hanyang University (HY-IACUC-2018-0148A).

### Bioinformatics analysis

Lung cancer patient data were obtained from an online database (https://software.broadinstitute.org/morpheus) and (www.kmplot.com) according to previous reports [29, 41]. To establish the clinical relevance of FoxM1 expression to the survival rates of lung cancer patients, the database, which was established using gene expression data and survival information of 1885 lung cancer patients, was used after excluding biased arrays. The expression values for FoxM1 and clinical data for those samples were extracted and used for survival analysis. The samples were split into high and low groups using FoxM1 expression.

The gene expression levels of PLK1 and FoxM1 were divided into low and high quartiles. Subsequently, a univariate Cox regression was performed to calculate the hazard ratio of the KM survival plot with 95% confidence intervals and log-rank *P* value. The calculations were performed using the R script. A* P* value < 0.05 was considered statistically significant. HR is the ratio of the hazard rates corresponding to the conditions described by two levels of an explanatory variable in a survival analysis.

### Transcriptome profiling

Total RNA was extracted from the indicated invasive cells and non-invasive cells expressing mock, wild-type, S25A, or S25E-FoxM1 using TRIzolTM reagent (Thermo Fisher Scientific) according to the manufacturers’ direction. For this, cells were seeded in the upper chamber filled with Matrigel (BD Biosciences). Fourteen days after seeding, trypsin was treated for collecting invasive cells attached to the outer surface and non-invasive cells attached to the inner well [29]. RNA purity and integrity were evaluated using an ND-1000 spectrophotometer (NanoDrop, Wilmington, DE, USA) and Agilent 2100 bioanalyzer (Agilent Technologies, Santa Clara, CA, USA). The Affymetrix whole transcript expression array process was executed according to the manufacturer's protocol (GeneChip Whole Transcript (WT) PLUS reagent kit). cDNA was synthesized using the GeneChip WT Amplification kit as described by the manufacturer. The sense cDNA was fragmented and biotin-labeled with terminal deoxynucleotidyl transferase using the GeneChip WT Terminal labeling kit. Approximately 5.5 μg of labeled DNA target was hybridized to the Affymetrix GeneChip Human 2.0 ST Array at 45 °C for 16 h. Hybridized arrays were washed and stained on a GeneChip Fluidics Station 450 and scanned on a GCS3000 Scanner (Affymetrix). Signal values were computed using the Affymetrix® GeneChip™ Command Console software.

### Microarray analysis

Raw data were extracted automatically using an Affymetrix data extraction protocol in the Affymetrix GeneChip® Command Console® software. After importing CEL files, the data were summarized and normalized with the robust multi-average (RMA) method implemented in the Affymetrix® Expression Console™ software. We exported the results of gene-level RMA analysis and performed a differentially expressed gene analysis. The analysis comparing invasive S25E-FoxM1 with non-invasive mock or invasive mock was carried out based on fold changes. For transcriptome data, gene probes with significant fold changes (more than 1.5) were clustered. To develop a significant probe list, we performed a gene-enrichment and functional annotation analysis using GO (http://geneontology.org/) and KEGG (http://kegg.jp). All statistical tests and visualizations of differentially expressed genes were conducted using R statistical language v. 3.1.2. (www.r-project.org).

### ELISA assay

To measure secreted cytokine levels from the THP-1 cells co-cultured with A549^S25E^ cells for 48 hours, enzyme-linked immunosorbent assay (ELISA) was performed, using commercially available kits, following the manufacturer's instructions using VEGF-A (Abcam; Cambridge, UK, Cat. No. ab119566) and TGF-β1 (Abcam; Cat. No. ab100647) markers. Standards and cultured medium from the THP-1 cells co-cultured with A549^S25E^ cells were added into a 96-well plate precoated with anti-VEGFA or anti-TGF-β1 antibodies and then incubated at room temperature (RT). After washing, biotinylated anti-VEGFA or anti-TGF-β1 antibodies were added and incubated at RT. Following washing, HRP-conjugated streptavidin was added to the wells and incubated at RT. After washing, a TMB substrate solution was added. After incubation at RT, stop solution was added to change the color. The color intensity was measured at 450 nm using an M4 microplate reader (Molecular Devices; San Jose, CA, USA).

### Immunofluorescence

A549 cells grown on coverslips were fixed with 4% paraformaldehyde. Methanol was used for permeabilization. Cells were washed three times with 0.1% Triton X-100 in PBS, incubated overnight at 4 °C in PBS containing 0.1% Triton X-100 (PBST) and 3% bovine serum albumin to block nonspecific interactions, and then incubated with anti-FoxM1 (Santa Cruz Biotechnology, Sc-271746) and RFP (Life Technologies, R10367). The cells were washed three times with PBST and then incubated with FITC-conjugated anti-mouse secondary antibodies (Jackson ImmunoResearch Laboratories, West Grove, PA, USA), Cy3-conjugated anti-rabbit secondary antibodies (Jackson ImmunoResearch Laboratories), and 4′, 6-diamidine-2-phenylindole (DAPI) (Sigma-Aldrich) for staining nuclear DNA. Images of cells were collected and evaluated with a confocal microscope FW3000 (Olympus; Tokyo, Japan).

### Immunohistochemistry

For immunohistochemistry, three types of LUAD and normal tissue specimens were analyzed (Super Bio Chips, Korea). Tumor tissue sections were deparaffinized and rehydrated with xylene and graded alcohol, respectively. The sections were permeabilized with PBST for 10 min at RT. To eliminate the activity of endogenous peroxidase, peroxide blocking solution was treated at room temperature for 10 min. After washing two times with PBS, the sections were incubated with anti-FoxM1 (Santa Cruz Biotechnology Inc.; 1:200) or anti-p-Serine (Santa Cruz Biotechnology Inc.; 1:200) antibodies overnight at 4℃. The tissues were washed three times with Tris buffered saline (TBS) and then incubated with FITC-conjugated anti-mouse secondary antibodies (Jackson ImmunoResearch Laboratories, West Grove, PA, USA; 1:200), Cy3-conjugated anti-rabbit secondary antibodies (Jackson ImmunoResearch Laboratories; 1:200) at 30 min at RT and 4′, 6-diamidine-2-phenylindole (DAPI) (Sigma-Aldrich) for staining nuclear DNA. For Ki67 staining, paraffin tissue sections of mouse lung tissues were used with anti-Ki67 antibody (Abcam; Cat. No. ab16667; 1:200). The tissues were washed three times with TBS and then incubated with FITC-conjugated secondary antibodies (Jackson ImmunoResearch Laboratories, West Grove, PA, USA) at 30 min at RT, and 4′, 6-diamidine-2-phenylindole (DAPI) (Sigma-Aldrich) for staining nuclear DNA. Images of cells were collected and evaluated with a confocal microscope FW3000 (Olympus; Tokyo, Japan).

### Chromatin immunoprecipitation assays

The ChIP assay was performed as described [30]. To examine the interaction between FoxM1 and the promoters for c-Fos, STAT1, IL6, VEGFA, IL1A, IL1B, IFITM1, and STING, A549 cells expressing wild-type, S25A, or S25E FoxM1 were used. Cross-linking was achieved with 1.4% formaldehyde. Cells were lysed with lysis buffer and chromatin was sheared by sonication and incubated with polyclonal antibodies to FoxM1 or normal IgG for 16 h. Sheared chromatin was incubated with protein A beads (Santa Cruz Biotechnology) for 3 h and washed five times with lysis buffer. Chelex 100 slurry (Bio-Rad; Hercules, CA, USA) was added to the washed beads, which were then boiled and incubated with Proteinase K (Invitrogen; Waltham, MA, USA) at 55 °C for 30 min. The samples were boiled again and cleared by centrifugation, and then the supernatants were collected for qRT-PCR. The bound chromatin fraction was amplified with promoter-specific primers flanking A/G-C/T-AAA-C/T-A boxes (FoxM1 binding sites) [42] for 50 cycles (Table S4). For the interaction between IRF3 and the promoters for IFITM1, specific anti-IRF3 antibody was used. The bound chromatin fraction was amplified with promoter-specific primers flanking GAAA-G/C-G/C-GAAA boxes (IRF3 binding sites) [43] for 50 cycles. Real-time PCR was carried out on a CFX96 Real-Time PCR system (Bio-Rad) using SYBR Green Master Mix (Bio-Rad, #1708880). The data were analyzed by the comparative C_T_(ΔΔC_T_) method. The relative occupancy of the immunoprecipitated factor at a locus was estimated using the following equation: 2^(Ct^IgG^ – Ct^target^), where Ct^IgG^ and Ct^target^ are the mean threshold cycles of PCR performed in triplicate on DNA samples from normal IgG and FoxM1 immunoprecipitations, respectively.

### Statistical analysis

All data are given as means ± SDs of at least three independent experiments, each performed in triplicate. Results were analyzed for statistically significant differences using Student's *t*-test or two-way ANOVA test, and statistical significance was set at *p* < 0.05 (*p < 0.05; **p < 0.01; ***p < 0.001; ^#^*p* < 0.05; ^##^*p* < 0.01; ^###^*p* < 0.001).

## Results

### Concurrent upregulation of FoxM1 and PLK1 correlated with poor survival of lung adenocarcinoma (LUAD) patients

**Fig. 1:**
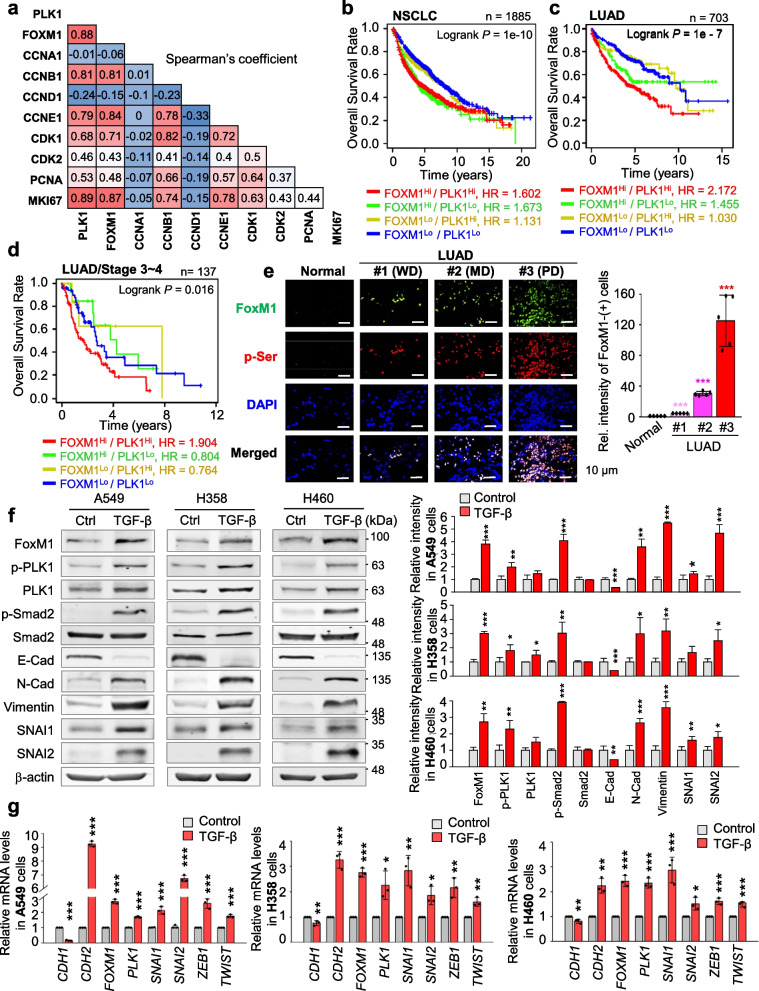
Concurrent upregulation of FoxM1 and PLK1 is correlated with poor survival of LUAD patients. **a** Analysis of Spearman’s coefficient for the correlations between cell cycle-regulatory factors including *PLK1*, *FOXM1*, *CCNA1*, *CCNB1*, *CCND1*, *CCNE1*, *CDK1*, *CDK2*, *PCNA*, and *MKI67*. **b-d** The overall survival (OS) of patients with non-small lung cancer (*n* = 1885) (**b**), lung adenocarcinoma (LUAD) (*n* = 703) (**c**), and stages 3–4 of LUAD (*n* = 137) (**d**) were analyzed according to *PLK1* and *FOXM1* expression levels using KM PLOTTER. High (Hi) and low (Lo) were generated by separating patients according to expression at the median cut-off. **e** Using human lung tissues from LUAD patients and normal individual, immunohistochemistry analysis was displayed with anti-FoxM1 (Green) and anti-p-Serinie (Red) antibodies. The relative intensity of cells that exhibited positive FoxM1 was analyzed and plotted. WD, well differentiated (grade 1); MD, moderately differentiated (grade 2); PD, poorly differentiated (Grade 3), n > 5000. *p < 0.05; **p < 0.01; ***p < 0.001. Data are presented as mean ± SD. **f** and **g** A549, NCI-H358 (H358), and NCI-H460 (H460) NSCLC cells treated with 5 ng/mL of TGF-β for 48 h. **f** Immunoblotting was performed to measure the expression and phosphorylation of PLK1 using specific antibodies for FoxM1, PLK1, p-PLK1^T210^, p-Smad2^S465/S467^, Smad2, E-cadherin, N-cadherin, vimentin, SNAI1, SNAI2, and β-actin in A549, NCI-H358 (H358), and NCI-H460 (H460) cells (left panel). The relative band intensities for FoxM1, p-PLK1^T210^, PLK1, p-Smad2.^S465/S467^, Smad2, E-cadherin, N-cadherin, vimentin, SNAI1, and SNAI2 were quantified using LI-COR Odyssey software (right panel). **g** qRT-PCR was performed for *CDH1*, *CDH2*, *FOXM1*, *PLK1*, *SNAI1*, *SNAI2*, *ZEB1*, and *TWIST* expression in A549 (left panel), NCI-H358 (middle panel), and NCI-H460 (right panel) cells. Data are presented as mean ± SD of at least three independent experiments (significantly different from the experimental control). *p < 0.05; **p < 0.01; ***p < 0.001

The majority of KEGG pathways in active PLK1-induced invasive NSCLC is related to ECM-adhesion and cell cycle-related factors [29] (Fig. S2a). Because proliferation is closely connected with metastasis [31], we investigated proliferating factors related to major metastatic drivers in PLK1-driven invasiveness [29]. Under conditions of low E-cadherin (*CDH1*) and high N-cadherin (*CDH2*), *TGFB1*, and *TGFB2* in active PLK1-expressed NSCLC cells, the expression of cell cycle factors including *FOXM1*, *CCNB1*, *CCNE1*, and *MKI67* was highly correlated with PLK1 expression (Fig. S2b). In mRNA expression, the correlation between *PLK1* and *FOXM1* (Spearman: 0.88, *p* = 1.68e-55; Pearson: 0.86, *p* = 3.22e-51) or *MKI67* (Spearman: 0.89, *p* = 3.67e-59; Pearson: 0.89, *p* = 1.13e-59) was positive compared with those of others, including *CCNA1* and *CCND1*, extracted from the cBio-Portal analysis in NSCLC patients (Fig. 1a, Fig. S2c-d, Table S5). Notably, both PLK1 and FoxM1 are regulators for EMT as well as mitosis [8–10]. For their clinical relationship, the overall survival (OS) corresponding to the expression level of each factor in NSCLC or LUAD was observed using a Kaplan–Meier (KM) plot database [41]. The clinical features of lung cancer patients were presented (Table S6). The OS with high *FOXM1/PLK1* expression was significantly shorter than that with low *FOXM1/PLK1* expression (NSCLC, *n* = 1885, HR = 1.602, log-rank *P* = 1e-10; LUAD, *n* = 703, HR = 2.172, log-rank *P* = 1e-7) (Fig. 1b-c, Table S6-S7). Survival until first progression (FPS) rates of patients with NSCLC or LUAD were correlated with *FOXM1/PLK1* expression, similar to the correlation with OS (NSCLC, *n* = 982, HR = 1.75, log-rank *P* = 1.2e-08; LUAD, n = 461, HR = 2.58, log-rank *P* = 5.3e-09; Fig. S2e-f). However, the OS or FPS rates of patients with lung squamous cell cancer (LUSQ) were not significant (Fig. S2g). In a clinical analysis of metastatic NSCLC or LUAD patients at TNM stage N1, the OS of patients with high *FOXM1/PLK1* expression was shorter than for those with low *FOXM1/PLK1* (Fig. S2h). In addition, clinical analysis of 137 advanced LUAD patients at stages 3–4 showed shorter OS rates of advanced patients with high *FOXM1/PLK1* expression than of those with low expression (Fig. 1d, *n* = 137, HR = 1.904, log-rank *P* = 0.016; Table S7). These data indicate that high expression of FoxM1 and PLK1 occurs concurrently with a poor prognosis for patients with primary and advanced NSCLC, especially LUAD.

To understand *FOXM1* expression in metastatic LUAD, data from TCGA were analyzed by stage in normal and tumor tissue, constructing a heatmap based on the degree of expression of *FOXM1* (Fig. S2i). In genomic analysis, *FOXM1* expression was much higher in the advanced stages 2–4 (81%; 22 tumors/27 total) than those of primary tumor stage 1 (70%; 17 tumors/24 total). The expression pattern of *FOXM1* was like that of other proliferation factors including *PLK1*, *CCNB1*, and *MKI67*. *SNAI1*, a mesenchymal marker, was upregulated compared with normal tissues in stage 2–4 LUAD patients having high *FOXM1* (Fig. S2i). Accordingly, the concurrent high expression of FoxM1 and PLK1 showed a high correlation with survival of primary and advanced NSCLC patients, particularly in LUAD but not in LUSQ.

To explore the phosphorylation and expression of FoxM1 in LUAD patients, we carried out immunohistochemistry on tissues from LUAD patients (Super Bio Chips, Korea), categorized by their tumor grade (Table S8). These tissues were subjected to staining with anti-FoxM1 and anti-p-Serine antibodies. FoxM1 and p-Serine were co-stained at the same location within LUAD tissues. Importantly, we observed a notable increase in the levels of both FoxM1 and p-Serine in LUAD patients compared to those from a healthy individual with normal lung tissues (Fig. 1e, Table S8). Furthermore, these elevated levels were even more pronounced in advanced LUAD tissues when compared to low-grade LUAD tissues.

### FoxM1 is phosphorylated by PLK1 through direct interaction in TGF-β -induced EMT

To investigate the functions of FoxM1 and PLK1 in the EMT, we observed the expression of FoxM1 and PLK1 in primary A549 and metastatic NCI-H358, NCI-H1299, and NCI-H460 NSCLC cells. Compared with normal lung epithelial BEAS-2B cells, the expression of FoxM1 and PLK1 was higher in NSCLC cells (Fig. S3a). The level of active PLK1 phosphorylated at Thr210 was higher in NSCLC cells than in normal BEAS-2B cells. Using previously published microarray data of lung cancer (GSE 114761), FoxM1 and PLK1 were upregulated in TGF-β-induced EMT in A549, NCI-H522, NCI-H1944, and NCI-H2122 cells (Fig. S3b). Additionally, changes of FoxM1 and PLK1 were observed in TGF-β-treated A549, NCI-H358, and NCI-H460 cells. Treatment with TGF-β, an inducer of the EMT, increased the expression of *CDH2*, a mesenchymal marker, but decreased the level of *CDH1*, an epithelial index, relative to those of control cells (Fig. 1f-g). Under these conditions, immunoblot analysis revealed that FoxM1 proteins increased approximately 3.8, 3.1, and 2.8 times in A549, NCI-H358, and NCI-H460 cells, respectively. This increase in FoxM1 levels coincided with the upregulation of p-Smad2, a downstream factor of TGF-β signaling pathway, induced by TGF-β treatment (Fig. 1f, Fig. S3c). In response to TGF-β treatment, the levels of total and the active form of PLK1 phosphorylated at Thr210 increased in TGF-β-treated NSCLC cells (Fig. 1f, Fig. S3c). qRT-PCR showed that *PLK1* and *FOXM1* levels increased by a factor of 2–3 compared with a control in TGF-β-treated NSCLC cells when the transcriptional factors for EMT including *SNAI1*, *SNAI2*, *ZEB1*, and *TWIST* increased (Fig. 1g). Therefore, active PLK1 and FoxM1 are concurrently upregulated in TGF-β-induced EMT of NSCLC.

**Fig. 2:**
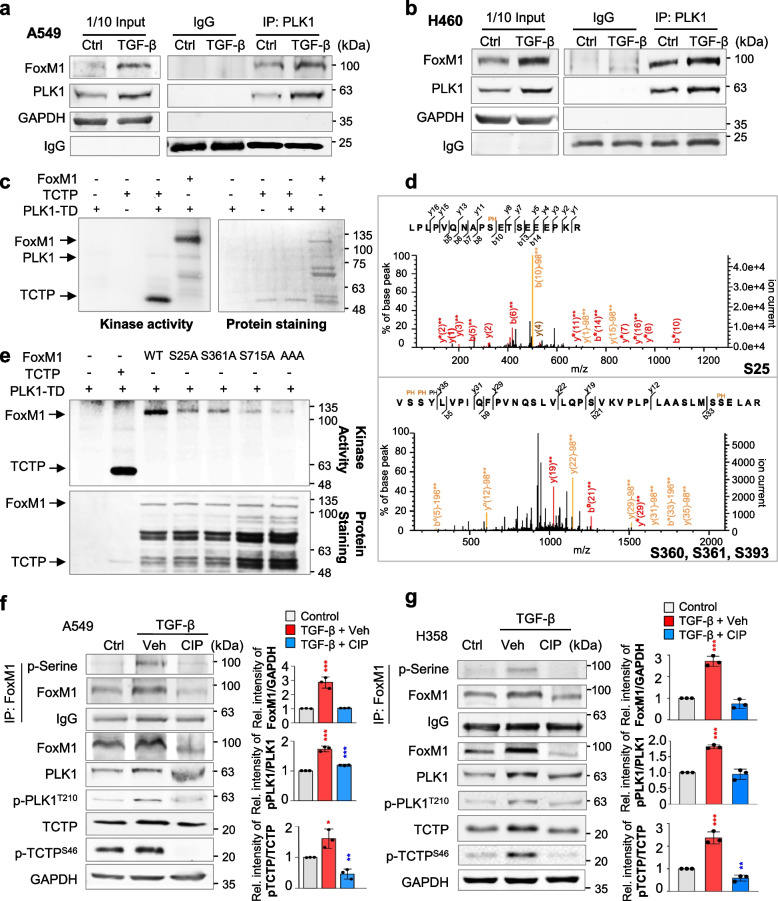
TGF-β-treated EMT results in phosphorylation of FoxM1 by PLK1 by direct interaction. **a**, **b** A549 (**a**) and NCI-H460 (**b**) were treated with TGF-β (5 ng/mL) for 48 h. Immunoprecipitation of cell lysates was performed with normal IgG or anti-PLK1 antibody and then immunoblotting was performed with anti-FoxM1 antibody. **c** An in vitro kinase assay was performed with an active version of PLK1 with T210D (PLK1-TD), radioactive ATP, and purified GST-FoxM1. GST-tagged TCTP was used as the positive control. **d** In LC–MS/MS analysis, possible phosphorylation residues of FoxM1 by PLK1 were newly detected at the S25, S360, S361, and S393 sites. **e**, Purified GST-tagged wild-type, S25A, S361A, S715A, and S25/S361/S715A (AAA) FoxM1 mutants were used for a PLK1 kinase assay with radioactive ATP. **f**, **g** Phosphorylation of FoxM1 in A549 (**f**) and NCI-H358 (**g**) cells treated with TGF-β for 48 h. Treatment with calf intestinal alkaline phosphatase (CIP) reduced the phosphorylation of FoxM1 and PLK1 in TGF-β-induced EMT. Immunoprecipitation was performed with anti-normal IgG (Fig. S4d-e) or anti-FoxM1 antibody, and then immunoblotting was conducted with anti-p-Serine antibodies. Immunoblotting was performed for FoxM1, PLK1, p-PLK1^T210^, TCTP, and p-TCTP^S46^ using specific antibodies. TCTP was used as a positive control of the PLK1 substrate. Data are presented as mean ± SD of at least three independent experiments (significantly different from the experimental control). *p < 0.05; **p < 0.01; ***p < 0.001

Because of upregulation of active PLK1 and FoxM1 in TGF-β-induced EMT (Fig. 1) and their functions as positive EMT regulators [9, 10, 36, 37], we hypothesized that FoxM1 and PLK1 work cooperatively in TGF-β-induced EMT. For this, immunoprecipitation assays were performed to observe their interaction in TGF-β-induced EMT. In TGF-β-treated A549 cells and NCI-H460 cells, endogenous PLK1 interacted with endogenous FoxM1 (Fig. 2a-b). Exogenously expressed FoxM1 also interacted with PLK1 in TGF-β-treated A549 cells (Fig. S4a). Thus, PLK1 and FoxM1 interact directly during TGF-β-induced EMT.

Since active PLK1 drives the EMT, to explore phosphorylation of sites of FoxM1 by PLK1 for EMT, in vitro PLK1 kinase assays and LC–MS/MS analyses were performed. In the tests, PLK1 strongly phosphorylated FoxM1 as much as did TCTP, a positive control (Fig. 2c). LC–MS/MS analysis illustrated newly predicted phosphorylation sites at the Ser25, Ser360, Ser361, and Ser393 residues (Fig. 2d). Non-phosphomimetic alanine substitutes of FoxM1 at these newly predicted phosphorylation sites and at previously found phosphorylation residues (Ser715 and Ser724) [28] were generated using site-directed mutagenesis. The in vitro PLK1 kinase assay showed that the band intensity of non-phosphomimetic alanine-single mutants at Ser25, Ser361, and Ser715 residues was markedly reduced compared with that of wild-type FoxM1 (Fig. 2e, Fig. S4b). Triple mutant (S25A/S361A/S715A; AAA) was barely phosphorylated by PLK1 in vitro PLK1 kinase assay (Fig. 2e, Fig. S4c). In addition to the previously found Ser715 residue, Ser25 and Ser361 residues of FoxM1 were potent phosphorylation sites by PLK1. To examine whether FoxM1 phosphorylation depends on the EMT, A549 and NCI-H358 cells were treated with TGF-β (Fig. 2f-g). Phosphatase treatment reduced the levels of p-FoxM1^Ser^, p-PLK1^T210^, and p-TCTP^S46^ and retarded the shifted bands of PLK1, TCTP, and FoxM1, which were upregulated by TGF-β treatment (Fig. 2f-g, Fig. S4d-e), implying that phosphorylation of FoxM1 by PLK1 occurs during the EMT in both primary A549 and metastatic NCI-H460 LUAD cells. These phosphorylation residues are evolutionarily conserved in several species (Fig. S4f).

### Phosphorylation of FoxM1 at Ser25 facilitates cell migration and invasiveness but not cell proliferation

**Fig. 3:**
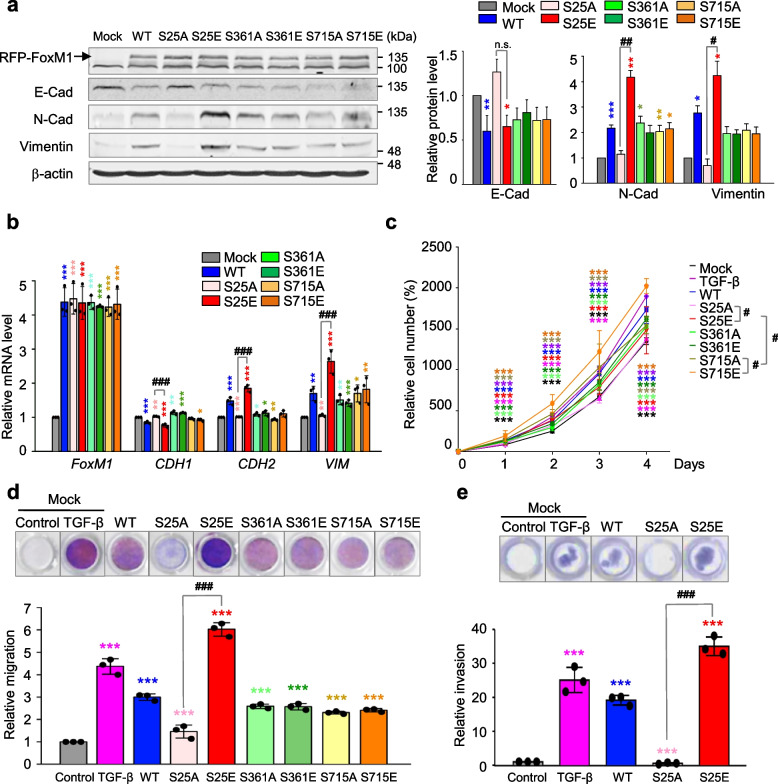
Phosphorylation of FoxM1 at Ser25 facilitates cancer cell migration and invasiveness but not cell proliferation. RFP-tagged wild-type (WT) FoxM1 and S25A, S25E, S361A, S361E, S715A, and S715E mutants were expressed in A549 cells. A549 cells were treated with doxycycline to express RFP-tagged FoxM1. **a** Immunoblotting was performed using specific antibodies for RFP, N-cadherin (N-Cad), E-cadherin (E-Cad), vimentin, and β-actin (left panel). The band intensity values were quantified using LI-COR Odyssey software, normalized, and plotted (right panel). **b** qRT-PCR was performed for *FOXM1*, *CDH1*, *CDH2*, and *VIM* in A549 cells expressing wild-type or mutants FoxM1. *p < 0.05; **p < 0.01; ***p < 0.001; (*n* = 3). Data are presented as mean ± SD. **c** Cell proliferation assay was performed (*n* = 3). Data are presented as mean ± SD of at least three independent experiments. *p < 0.05; **p < 0.01; ***p < 0.001 compared with experimental control; ^#^*p* < 0.05 compared with indicated groups of cells. **d** Cells expressing wild-type or mutants of FoxM1 were subjected to a Transwell migration assay. As a positive control for migration, cells were treated with TGF-β. Three days after seeding, the cells on the bottom surface were stained with 0.05% crystal violet dye. Images of the Transwell cell migration assay were collected and analyzed with an Odyssey infrared imaging system and plotted. **e** An invasion assay was performed using A549 cells expressing wild-type or mutants of FoxM1. Seven days after seeding, the cells that invaded the bottom surface were stained with 0.05% crystal violet dye, and the relative absorbance was plotted. Data are presented as mean ± SD of at least three independent experiments (significantly different from the experimental control). *p < 0.05; **p < 0.01; ***p < 0.001 compared with experimental control. ^#^*p* < 0.05; ^##^*p* < 0.01; ^###^*p* < 0.001 compared with S25A FoxM1

We then studied functions of p-FoxM1 for the EMT. The phosphomimetics and non-phosphomimetics of FoxM1 were expressed in primary LUAD A549 cells, using a doxycycline-inducible expression system and site-specific mutagenesis to replace the serine residues with alanine for non-phosphomimetics and with glutamic acid for phosphomimetics. Under conditions with similar expression of different versions of FoxM1, the expression of S25E FoxM1 (FoxM1^S25E^) increased protein and mRNA levels of mesenchymal markers N-cadherin and vimentin, but the expression of expressing S25A FoxM1 (FoxM1^S25A^) did not (Fig. 3a-b, Fig. S1), indicating that expression of FoxM1^S25E^ induces the mesenchymal transition from epithelial cells. However, cells expressing phosphomimetics and non-phosphomimetics at Ser361 and Ser715 did not show differences in the levels of N-cadherin or vimentin. To understand the proliferation effects of each version of FoxM1, a cell proliferation assay was performed (Fig. 3c). A549 cells expressing FoxM1^S715E^ showed the highest proliferation effects, as reported previously for mitosis [44]. However, cells expressing phosphomimetics and non-phosphomimetics at the Ser25 and Ser361 residues were similar in terms of proliferation rate, indicating that phosphorylation at Ser25 and Ser361 of FoxM1 does not foster cell proliferation.

Next, we explored phosphorylation sites of FoxM1 related to the EMT. For this, a Transwell cell migration assay and an inverted invasion assay were performed in A549 cells expressing phosphomimetic and non-phosphomimetic FoxM1 (Fig. 3d-e, Fig. S1). TGF-β treatment increased cell migration and invasiveness approximately 5- and 25-fold compared with the control, respectively. The expression of FoxM1^S25E^ upregulated cell migration and invasiveness approximately 6- and 35-fold compared with the mock system, respectively. Cell migration and invasiveness were lower in cells expressing FoxM1^S25A^ than FoxM1^WT^. However, migration of cells expressing phosphomimetics and non-phosphomimetics at Ser361 and Ser715 residues was similar (Fig. 3d), indicating no relation of the function of phosphorylation at Ser361 and Ser715 of FoxM1 with the EMT. These patterns were consistent in LUAD HCC827 cells expressing FoxM1^S25E^ (Fig. S5a) with higher *CDH2* and lower *CDH1* than those of mock or FoxM1^WT^ and higher invasion approximately eightfold compared with the control (Fig. S5b). Therefore, FoxM1 phosphorylation at Ser25 enhanced cell migration and invasion but not cell proliferation.

### Phosphorylation of FoxM1 at Ser25 facilitates metastasis in a tail-vein injection mouse model

**Fig. 4:**
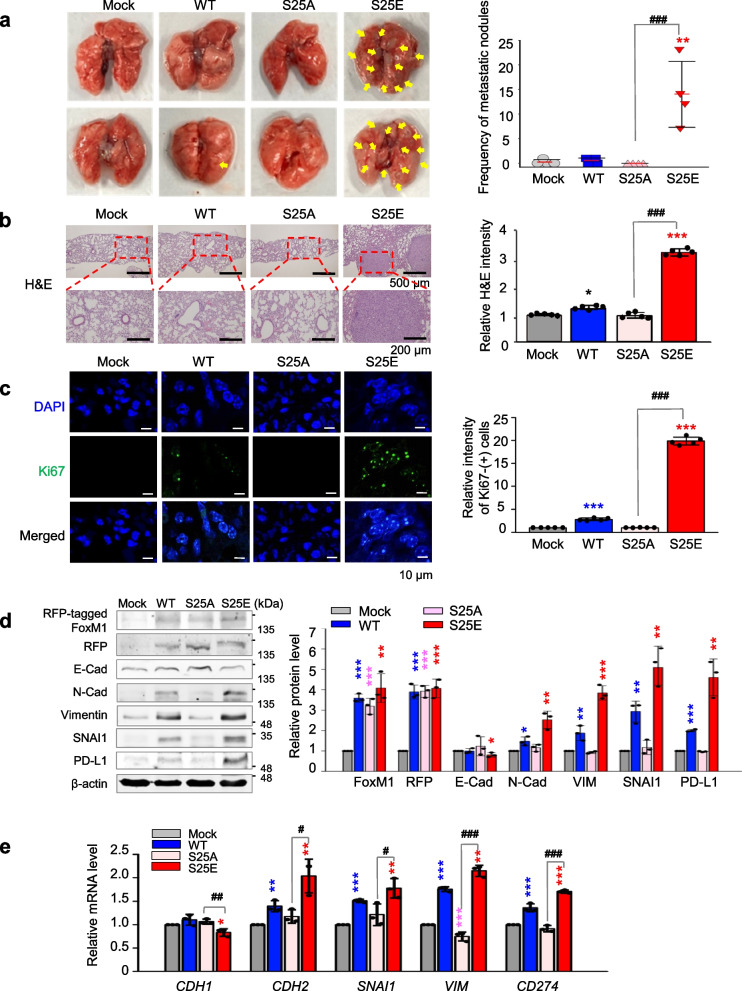
Phosphorylation of FoxM1 at Ser25 facilitates metastasis of NSCLC in a tail-vein injection model. A549 cells expressing RFP-tagged WT, S25A, and S25E of FoxM1 were injected intravenously into the tail-veins of four-week-old BALB/c nude mice, and the tumorigenic and metastatic properties were evaluated after 15 weeks. **a** Representative lung tumor from the mouse model (left panel). The number of metastatic lung tumors was counted and plotted. (*n* = 4 or 5) (right panel). Data are presented as mean ± SD. **b**, **c** Representative H&E staining (**b**, left panel) and Ki-67 staining (**c**, left panel) were performed using lung tissue from the mice.The relative density of H&E staining (**b**, right panel) and Ki-67-positive cells (**c**, right panel) was analyzed and plotted. *p < 0.05; **p < 0.01; ***p < 0.001. Data are presented as mean ± SD. **d** Immunoblotting was performed using lung tissue lysates from each mouse model. FoxM1, RFP, E-cadherin, N-cadherin, vimentin, SNAI1, PD-L1, and β-actin were detected using specific antibodies (left panel). The band intensity values were quantified using LI-COR Odyssey software, normalized, and plotted (right panel). **e** qRT-PCR was performed for *CDH1*, *CDH2*, *SNAI1*, *VIM*, and *CD274* using lung tissue lysates from each mouse model. The relative mRNA expression was plotted. *p < 0.05; **p < 0.01; ***p < 0.001 compared with experimental control. ^#^*p* < 0.05; ^###^*p* < 0.001 compared with S25A FoxM1

To assess whether phosphorylated FoxM1 at Ser25 triggers metastasis in vivo, A549 cells expressing wild-type (A549^WT^) or phospho-mutants FoxM1 at Ser25 (A549^S25E^, A549^S25A^) were injected into the tail vein of BALB/c nude mice (*n* ≥ 4) (Fig. S1). In mice injected with A549^S25E^ cells, the frequency of metastatic nodules in the lung was approximately 15 × that of mock (A549^Mock^) or A549^WT^ (Fig. 4a, right panel). Using mouse lung tissues, H&E staining of cells was higher in tissues injected with A549^S25E^ cells than in those injected with A549^Mock^, A549^WT^, or A549^S25A^ (Fig. 4b, right panel). Similarly, Ki67 staining revealed that Ki67-positive cells from mouse lung tissues injected with A549^S25E^ cells were 20 × higher than those of A549^Mock^ or A549^S25A^ (Fig. 4c, right panel). Based on H&E-positive or Ki67-positive cells, lung tissues from mice injected with A549^S25E^ cells showed tumor formation (Fig. 4b-c). Even in mice injected with A549^S25A^ cells, metastatic nodules and proliferation were not found, indicating that phosphorylation of FoxM1 at Ser25 is important in lung metastasis.

Immunoblotting using the lung tissues of mice showed that the levels of N-cadherin, vimentin, and SNAI1 were upregulated in tissues expressing FoxM1^S25E^ compared with the mock system (Fig. 4d). However, E-cadherin level was lower in tissues expressing FoxM1^S25E^ than FoxM1^Mock^, FoxM1^WT^, or FoxM1^S25A^. Consistent with immunoblotting, qRT-PCR analysis showed that the levels of *CDH2*, *SNAI1*, and *VIM* were approximately 2.5 × higher in lung tissues injected with A549^S25E^ than with A549^Mock^ (Fig. 4e), indicating that tissues expressing FoxM1^S25E^ underwent EMT and metastasis. The upregulated mesenchymal factors *CDH2*, *SNAI1*, and *VIM* in A549^S25E^ were downregulated by treatment with thiostrepton, a FoxM1 inhibitor [45] (Fig. S5c). The level of PD-L1 (encoded by *CD274*) was higher in tissues having A549^S25E^ than A549^Mock^, A549^WT^, or A549^S25A^ (Fig. 4d-e). Thus, p-FoxM1^Ser25^ enhances metastatic lung nodule formation through activation of the EMT and immune escape in an in vivo mouse model.

### Interferon signaling is activated in invasive cells with phosphorylated FoxM1

**Fig. 5:**
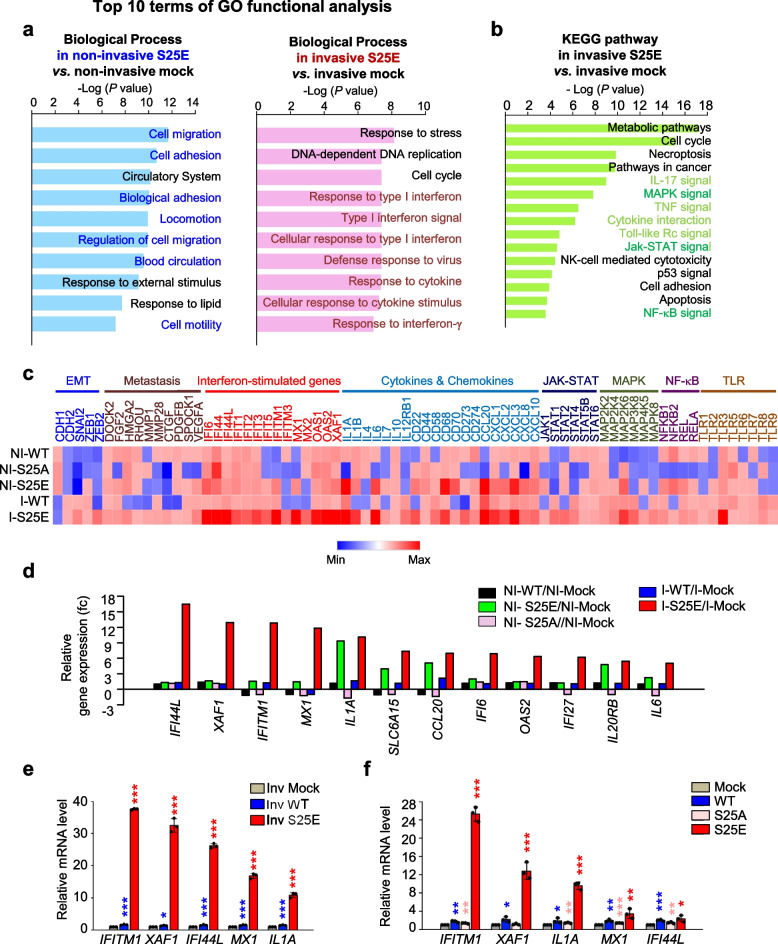
Interferon signaling is mainly activated in invasive cells having phosphorylated FoxM1 at Ser25. **a**, **b** Using non-invasive and invasive A549 cells expressing S25E of FoxM1, transcriptome profiles were analyzed by microarray. The transcriptome data were clustered by gene probes with fold change > 1.5 in cells expressing S25E FoxM1. Gene Ontology (GO) analysis of transcriptome profiles was performed as biological processes (**a**) and KEGG pathways (**b**). KEGG pathways were analyzed, and the signaling pathways had higher gene numbers in cells expressing invasive S25E than in those expressing mock. **c** Transcriptome comparison between gene profiles of invasive and non-invasive A549 cells expressing WT, S25A, and S25E. The MORPHEUS program was used to visualize the expression levels of genes related to the EMT, metastasis, interferon-stimulated genes, cytokines and chemokines, JAK-STAT, MAPK, NF-kB, and TLR signaling in non-invasive and invasive A549 cells expressing WT, S25A, and S25E of FoxM1. **d** Relative gene expression profile of the top 12 genes in invasive and non-invasive A549 cells expressing WT, S25E, and S25A FoxM1. **e**, **f** qRT-PCR was performed for the top five genes *IFITM1*, *XAF1*, *IF44L*, *MX1*, and *IL1A* in invasive A549 cells expressing FoxM1 (**e**) and total A549 cells expressing FoxM1 (**f**). *p < 0.05; **p < 0.01; ***p < 0.001; (*n* = 3). Data are presented as mean ± SD

For exploring pathways and factors triggered by p-FoxM1^Ser25^ depending on invasiveness, microarray analysis was performed. When invasive cells adhered to the external surface of the Transwell chamber filled with Matrigel, non-invasive cells adhered to the inner side of the well. Notably, A549^S25A^ cells were never exhibited invasion. Using invasive and non-invasive cells expressing FoxM1^S25E^ or FoxM1^WT^, transcriptome profiles (Supplementary Dataset 1) were analyzed with a microarray (Fig. 5). In Gene Ontology (GO) analysis of transcriptome profiles of non-invasive A549^S25E^ cells compared with non-invasive A549^Mock^ cells, genes related to cell migration, adhesion, and the circulatory system were significantly changed in the top three terms of the GO functional analysis, indicating that non-invasive A549^S25E^ cells experienced changes in the levels of genes related to intravasation (Fig. 5a, left panel). After invasion, invasive A549^S25E^ cells showed changes in genes related to interferon signaling and cytokines in the top 10 terms of the GO analysis compared with invasive mock cells (Fig. 5a, right panel). KEGG pathways were analyzed using significantly changed genes having a fold change cutoff ≥ 1.5 (Fig. 5b).Several inflammatory pathways, including IL17, MAPK, TNF, cytokine interaction, Toll-like receptor (TLR), JAK-STAT, and NF-κB signaling, were involved in invasive A549^S25E^ cells relative to A549^Mock^. The highly expressed genes including interferon-stimulated genes (ISGs) in the microarray were displayed in a heatmap (Fig. 5c). The levels of genes related to EMT, metastasis, cytokines, ISGs, JAK-STAT, MAPK, and NF-κB signaling were highly upregulated in invasive A549^S25E^ cells compared with A549^Mock^. Inflammatory cytokines such as *IL1A*, *IL1B*, and *IL6* were highly expressed in invasive A549^S25E^ cells (Fig. 5c), consistent with the increase of cytokine stimuli and responses observed in the biological process (Fig. 5a, right panel) and cytokine interaction detected in the KEGG pathway (Fig. 5b). The relative gene change analysis revealed that *IFI44L*, *XAF1*, *IFITM1*, *MX1*, and *IL1A* were the top five most highly expressed of the invasive A549^S25E^ cells among the 53,617 genes analyzed (Fig. 5d). Using qRT-PCR, the expression of *IFI44L*, *XAF1*, *IFITM1*, *MX1*, and *IL1A* was reanalyzed in invasive A549 cells (Fig. 5e) and total A549 and HCC827 cells (Fig. 5f, Fig. S5d). *IFITM1* was the most highly expressed among invasive cells or total cells expressing FoxM1^S25E^. *IL1A* was upregulated in both non-invasive and invasive A549^S25E^ cells (Fig. 5d) and was the third most highly expressed gene in the total A549^S25E^ cells. Therefore, ISGs and *IL1A* were activated in invasive cells expressing phosphomimetic FoxM1.

### p-FoxM1^S25^ triggers cytokine expression for recruitment of macrophages and polarization of M2d-TAM

**Fig. 6:**
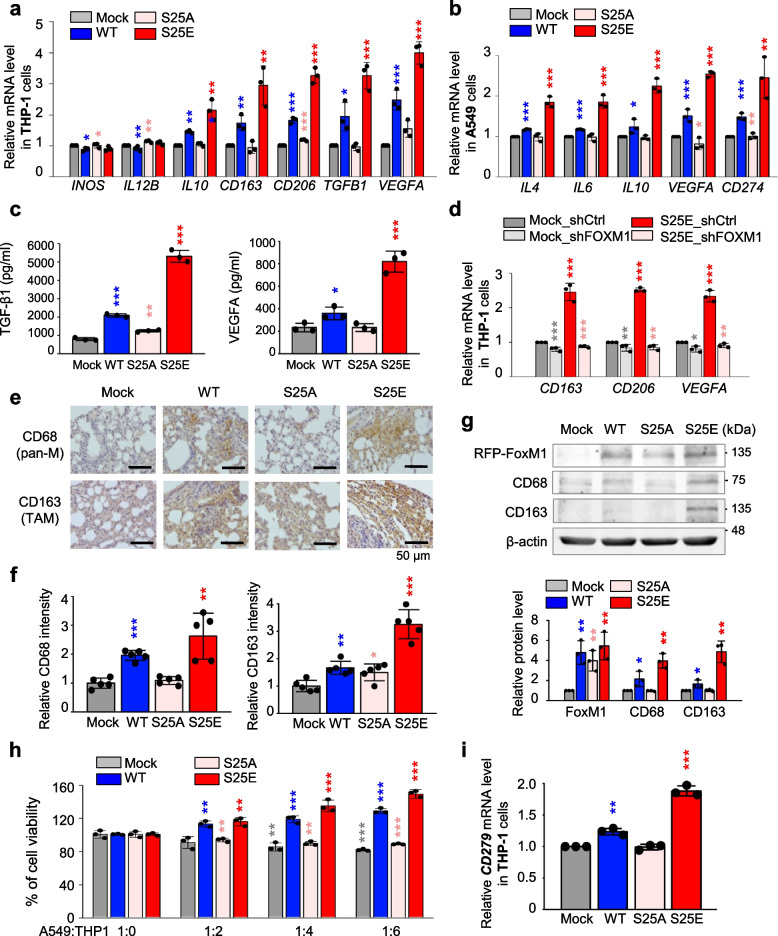
p-FoxM1.^S25^ functions in recruitment of macrophages and triggers polarization of M2-like TAM. **a**, **b** Monocyte THP-1 cells were co-cultured with A549 cells expressing mock, WT, S25A, and S25E FoxM1 for 48 h. In THP-1 cells, qRT-PCR was performed for markers of M1 (*INOS*, *IL12B*), M2 (*IL10*, *CD163*, *CD206*), and TAM (*TGFB1*, *VEGFA*) (**a**). In A549 cells, qRT-PCR was performed for *IL4*, *IL6*, *IL10*, *VEGFA*, and *CD274* (**b**). *p < 0.05; **p < 0.01; ***p < 0.001; (*n* = 3). Data are presented as mean ± SD. **c**, THP-1 cells were co-cultured with A549 cells expressing mock, WT, S25A, and S25E FoxM1. The secreted levels of TGF-β1 and VEGFA from THP-1 cells co-cultured with A549 cells were detected using ELISA. **d** Monocyte THP-1 cells were co-cultured with A549 cells expressing mock or S25E depleted FoxM1 using shRNA for 48 h. Using THP-1 cells, qRT-PCR was performed for *CD163*, *CD206*, and *VEGFA*. *p < 0.05; **p < 0.01; ***p < 0.001; (*n* = 3). **e**, **f** Representative CD68 (pan-macrophage marker) staining (**e**, upper panel) and CD163 (TAM marker) staining (**e**, lower panel) were performed using lung tissue from mice. The relative density of CD68 staining (**f**, left panel) and CD163 staining (**f**, right panel) was analyzed and plotted. *p < 0.05; **p < 0.01; ***p < 0.001. Data are presented as mean ± SD. **g** Immunoblotting was performed using lung tissue lysates from each mouse model. FoxM1, CD68, CD163 and β-actin were detected using specific antibodies (upper panel). The relative protein intensities were analyzed and plotted (lower panel). **h** The viability of A549 cells expressing mock, WT, S25A, and S25E FoxM1 was measured when the cells were co-cultured with monocyte THP-1 cells. The ratio between A549 cells and THP-1 cells was 1:0, 1:2, 1:4, and 1:6, as indicated. **i** Monocyte THP-1 cells were co-cultured with A549 cells expressing mock, WT, S25A, and S25E FoxM1. qRT-PCR was performed for *CD279* mRNA level in THP-1 cells. Data are presented as mean ± SD of at least three independent experiments (significantly different from the experimental control). **p < 0.01; ***p < 0.001; (*n* = 3)

Because FoxM1 is involved in alterations of macrophages [46, 47] and cytokines for macrophages activation are highly expressed in invasive A549^S25E^ cells (Fig. 5), we investigated whether p-FoxM1^Ser25^ is involved in macrophage polarization of the TME. Expression of the markers of M1 and M2 subtypes was observed in co-cultured human THP-1 and human monocyte-like cells derived from human pluripotent stem cells by qRT-PCR. The levels of M1 markers, *INOS* and *IL12B*, did not change significantly in THP-1 cells co-cultured with A549^S25E^ cells (Fig. 6a, Fig. S1). However, the levels of M2 markers *IL10*, *CD163*, and *CD206* increased up to 2.2, 3.0, and 3.2 times, respectively, in THP-1 cells co-cultured with A549^S25E^ cells compared with A549^Mock^ cells. Because TAMs have characteristics of pro-tumorigenesis and angiogenesis in the TME by release of TGF-β and VEGF [48, 49], the expression of *TGFB1* and *VEGFA* was tested (Fig. 6a). The levels of *TGFB1* and *VEGFA* were 3.2- and 4.0-fold higher, respectively, in THP-1 cells co-cultured with invasive A549^S25E^ cells compared with A549^Mock^. IL4 and IL10 are universal inducers for M2 type, and IL6 and VEGFA are inducers for M2d [48, 50]. Upregulation of cytokines for inducing M2d subtype, *IL4*, *IL6*, *IL10*, and *VEGFA*, was observed in A549^S25E^ cells compared with A549^Mock^ (Fig. 6b). This phenomenon was similar in primary human macrophages derived from human pluripotent stem cells co-cultured with invasive A549^S25E^ cells (Fig. S6a). In addition, upregulation of cytokines for inducing M2d subtype, *IL4*, *IL6*, *IL10*, and *VEGFA*, was observed in A549^S25E^ cells compared with A549^Mock^ cocultured with primary human macrophages (Fig. S6b). Furthermore, the secreted levels of TGF-β1 and VEGFA from the medium of THP-1 cells co-cultured with A549^S25E^ cells (Fig. 6c) or HCC827^S25E^ cells (Fig. S6c), determined by ELISA assay, increased up to 6.6 × and 3.5 × in A549^S25E^ cells, or 5.5 × and 2.0 × in HCC827 ^S25E^ cells, respectively, compared with mock cells (Fig. 6c).

To determine if the presence of FoxM1 is required in TAM differentiation, FoxM1 was depleted using shRNA targeting human FoxM1 (Fig. S6d). After FoxM1-shRNA was treated in A549^Mock^ (A549^Mock_shFOXM1^) or A549^S25E^ (A549^S25E_shFOXM1^) (Fig. S6e), THP-1 cells were co-cultured (Fig. 6d). The expression of *CD163*, *CD206*, and *VEGFA* was higher in THP-1 co-cultured with A549^S25E_shControl^ cells than with A549^Mock_shControl^ cells. However, when FoxM1 was depleted, the levels of *CD163*, *CD206*, and *VEGFA* were downregulated in THP-1 cells co-cultured with A549^S25E_shFOXM1^ cells compared with the control (Fig. 6d). Therefore, upregulation of cytokines such as IL4, IL6, and IL10 in invasive A549^S25E^ cells facilitated polarization of the M2d subtype of macrophage, which was eliminated by depletion of FoxM1 in the cocultured system.

Then, to investigate whether macrophages were recruited in TME by A549^S25E^ cells showing high expression of *VEGFA* [50] and *IL1A* [51], mouse tumor tissues were stained with specific macrophage antibodies for TAM marker CD163 and pan-macrophage marker CD68. Consistently, the relative CD68-positive intensity was ~ 2.6 × higher in lung tissues from mice injected with A549^S25E^ cells than with A549^Mock^ cells (Fig. 6e-f). The relative intensity of CD163-positive cells was 3.2 × higher in lung tissues from mice injected with A549^S25E^ cells than with A549^Mock^ cells (Fig. 6f). Immunoblotting using tissues of mice injected with A549^S25E^ cells revealed that CD163 and CD68 were highly expressed in TME of A549^S25E^ (Fig. 6g). Accordingly, macrophages were recruited and TAMs were induced in the TME of lung tissues from mice injected with LUAD expressing FoxM1^S25E^. We also investigated whether inhibition of TGF-β signaling reduced the levels of mesenchymal markers and IL6. Treatment of TGF-β-specific inhibitor SB431542 reduced the levels of p-PLK1, FoxM1, IFITM1, p-Smad2/3, IL6, IL1A, N-cadherin, SNAI1, and vimentin (Fig. S7), indicating that TGF-β signaling is important to FoxM1 phosphorylation-mediated downstream events including EMT and cytokine secretion for TAM polarization (Fig. S7).

PD-1 expression by TAMs regulates tumor immunity [52]. The higher level of *CD274* (encoding PD-L1, a ligand of PD-1) in mouse lung tissues injected with A549^S25E^ or in A549^S25E^ cells co-cultured with THP-1 or primary human macrophage cells was higher than that with A549^Mock^, A549^WT^, or A549^S25A^ cells (Fig. 4e, Fig. 6b, Fig. S6b, Fig. S6f), indicating viability of A549^S25E^ cells co-cultured with macrophages. The viability of A549^S25E^ cells increased at a ratio of 1:6 (A549:THP-1 cells) compared with A549^Mock^ cells (Fig. 6h). The increase in LUAD cell viability may be attributed to the potential interaction between PD-L1 of A549^S25E^ and PD-1 of THP-1 cells based on the previous studies [53] (Fig. 6h-i). Therefore, PD-L1 upregulation induced by FoxM1^S25E^ would evade the immune checkpoint by polarization of M2d-TAM that expressed PD-1 in the TME.

### p-FoxM1^S25^ directly activates the expression of IL1, IL6, VEGFA, SNAI1, and PD-L1 but not IFITM1 that are regulated by STING/TBK1/IRF3, JAK1/STAT1, and/or MEK signaling

**Fig. 7:**
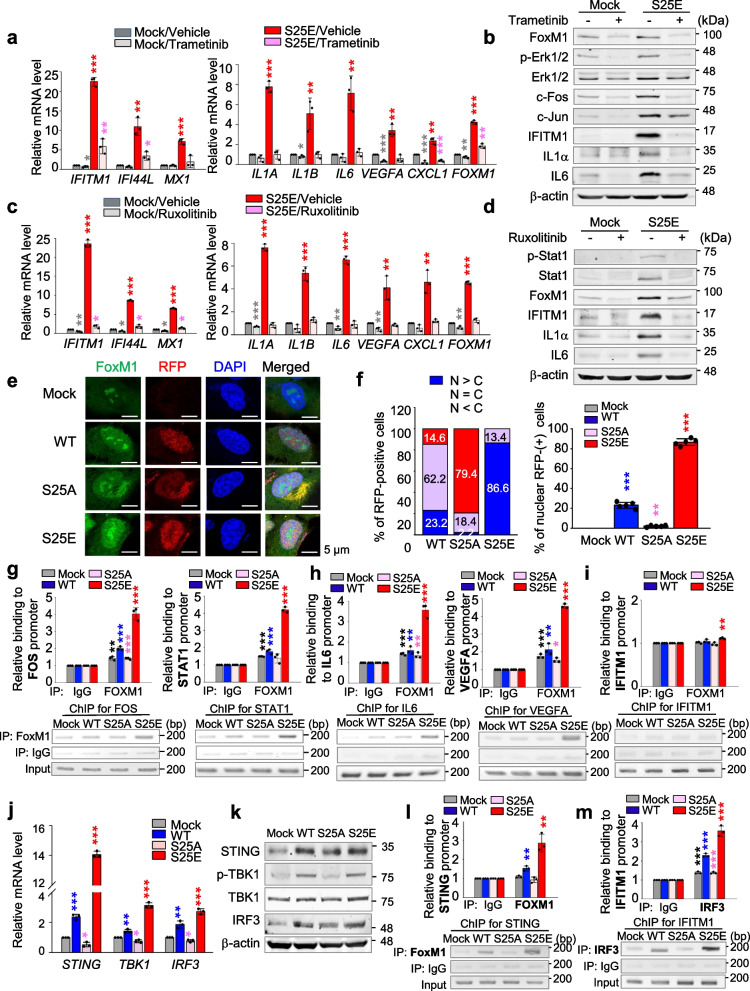
p-FoxM1^S25^ translocates to the nucleus and activates genes for monocyte recruitment, TAM polarization, immune escape, and angiogenesis by direct activation. **a**,** b** A549^S25E^ were treated with 1 μM trametinib, an inhibitor of MEK, for 48 h. **a** qRT-PCR was performed for interferon-stimulated genes (*IFITM1*, *IF44L*, and *MX1*), *IL1A*, *IL1B*, *IL6*, *VEGFA*, *CXCL1*, and *FOXM1* in A549^S25E^ cells. **b** Immunoblot analyses were performed using specific antibodies for FoxM1, p-Erk1/2, Erk1/2, c-Fos, c-Jun, IFITM1, IL1A, IL6, and β-actin. **c**,** d** A549^S25E^ cells were treated with 15 μM ruxolitinib, a JAK inhibitor, for 48 h. **c** qRT-PCR was performed for *IFITM1*, *IF44L*, *MX1*, *IL1A*, *IL1B*, *IL6*, *VEGFA*, *CXCL1*, and *FOXM1* in A549^S25E^ cells. **d** Immunoblot analyses were performed using anti-FoxM1, anti-STAT1, anti-p-STAT1, anti-IFITM1, anti-IL1A, anti-IL6, and anti-β-actin. **e** Immunofluorescence was performed with A549 cells expressing WT, S25A, or S25E mutant of FoxM1. FoxM1 (green), RFP (red), and DNA (DAPI, blue) was displayed. Scale bar, 5 μm. **f** The quantification of the population of cells in the cytoplasm, nucleus, and both is presented on the left. The percentage of cells that exhibited positive RFP (red) staining was assessed with the following categories (N > C, RFP staining predominantly in the nucleus; *N* = C, similar RFF levels in both the nucleus and cytoplasm; N < C, RFP staining mainly in the cytoplasm). The population of RFP-positive cells specifically in the nucleus was plotted (right). n > 800. **g-i** ChIP assays for FoxM1 binding to the promoters of *FOS* (**g**, left), *STAT1* (**g**, right), *IL6* (**h**, left), *VEGFA* (**h**, right), and *IFITM1* (**i**). Assays were performed on chromatin fragments using antibody to FoxM1 and normalized to pre-immune normal IgG. Immunoprecipitated fractions were assayed by qRT-PCR for binding the promoters of *FOS*, *STAT1*, *IL6*, *VEGFA*, and *IFITM1*. The qRT-PCR products were visualized in agarose-gel. **j **qRT-PCR was performed for *STING*, *TBK1*, and *IRF3* in A549^S25E^ cells. **k** Immunoblot analyses were performed using specific antibodies for STING, p-TBK1, TBK1, IRF3 and anti-β-actin. **l** ChIP assays were performed for FoxM1 binding to the promoters of *STING* in A549^S25E^ cells. **m** ChIP assays for IRF3 binding to the promoters of *IFITM1* in A549.^S25E^ cells. Assays were performed on chromatin fragments using antibody to IRF3 and normalized to pre-immune normal IgG. Immunoprecipitated fractions were assayed by qRT-PCR for binding the promoters of *IFITM1*. Data are presented as mean ± SD of three independent experiments (significantly different from the experimental control). *p < 0.05; **p < 0.01; ***p < 0.001; (*n* = 3)

ISGs including *IFITM1*, *IFI44L*, and *MX1* were highly expressed in invasive A549^S25E^ cells compared with the control (Fig. 5). ISGs were induced by several signaling pathways, including MAPK, JAK-STAT, and NF-κB signaling [54], which were detected in GO analysis (Fig. 5b) and APPYTER analysis (Fig. S8a-b) of the microarray data of invasive A549^S25E^. To elucidate the signaling pathway for expression of *IFITM1* in invasive A549^S25E^ cells, trametinib, ruxolitinib, fludarabine, and BI605906 were applied for inhibiting MEK, JAK1, STAT1, and IKKβ, respectively (Fig. 7a-d, Fig. S9a-d). The levels of *IFITM1*, *IFI44L*, and *MX1* were markedly downregulated by treatment with trametinib, ruxolitinib, and fludarabine but not by BI605906 in A549^S25E^ cells, while treatment with trametinib, ruxolitinib, fludarabine, and BI605906 markedly reduced the mRNA levels of *IL1A*, *IL1B*, *VEGFA*, and *IL6* for macrophage recruitment or TAM polarization [51] (Fig. 7a and c, Fig. S9a and S9c). Moreover, the levels of *CDH2*, *VIM*, and *SNAI1/2* were downregulated by treatment with trametinib, ruxolitinib, and fludarabine, but not by treatment with BI605906 (Fig. S9e-h). Thus, ISGs and mesenchymal factors upregulated in A549^S25E^ can be activated by MEK and JAK1/STAT1 pathways but not by NF-kB.

For further investigation of whether p-FoxM1^S25^ upregulates its transcriptional activity, the nuclear location of FoxM1^S25E^ was observed by immunostaining (Fig. 7e-f, Fig. S10). Up to 86.6% of RFP-tagged FoxM1^S25E^ was located in the nucleus, while only 2.2% of FoxM1^S25A^ was located in the nucleus (Fig. 7e-f, Fig. S10), indicating that FoxM1^S25E^ dominantly translocates into the nucleus. To determine whether p-FoxM1^S25^ can directly bind the promoter regions of genes for proinflammation, EMT, immune escape, or ISGs, conserved binding sequences around promoter regions of these genes were analyzed (Fig. S11a). A ChIP assay using anti-FoxM1 antibody revealed that FoxM1^S25E^ directly bound the promoters of *FOS*, *STAT1*, *IL6*, *VEGFA*, *IL1A*, *IL1B*, *SNAI1*, and *CD274* and upregulated their expression by 3–4 × compared with mock (Fig. 7g-h, Fig. S11b-e). However, FoxM1 did not bind to the promoter of *IFITM1* (Fig. 7i). Thus, p-FoxM1^S25^ directly activates the expression of genes for monocyte recruitment, TAM polarization, angiogenesis, immune escape, and EMT.

Next, we wanted to investigate which factors regulate the expression of *IFITM1*. Because STING-TBK1-IRF3 signaling is a well-established upstream regulator of IFN genes to mediate immune defense [55], the levels of STING, TBK1, and IRF3 was observed in A549^S25E^ cells. qRT-PCR analysis revealed that the expressions of *STING*, *TBK1*, and *IRF3* were highly upregulated in A549^S25E^ cells compared with A549^Mock^ cells (Fig. 7j). In addition, the levels of active p-TBK1 increased in A549^S25E^ cells compared with A549^Mock^ cells by immunoblot analysis (Fig. 7k). A ChIP assay using anti-FoxM1 antibody revealed that FoxM1^S25E^ directly bound the promoter of *STING* and upregulated the expression of STING by 3 × in A549^S25E^ cells compared with A549^Mock^ (Fig. 7l, Fig. S11f). Additional ChIP analysis using anti-IRF3 showed that IRF3 bound to the promoter regions of *IFITM1* and upregulated the expression of *IFITM1* by 4 × in A549^S25E^ cells compared with A549^Mock^ cells (Fig. 7m, Fig. S11g), indicating that FoxM1^S25E^ upregulates the expression of *STING*, which triggers the expression of TBK1 and IRF3. Consequently, IRF3 activates the expression of *IFITM1* in cells expressing FoxM1^S25E^. Therefore, the expression of *IFITM1* is regulated through the STING-TBK1-IRF3 pathway that is activated by p-FoxM1^S25^.

### IFITM1 induces invasiveness and TAM polarization in invasive FoxM1- or TGF-β-induced EMT

**Fig. 8:**
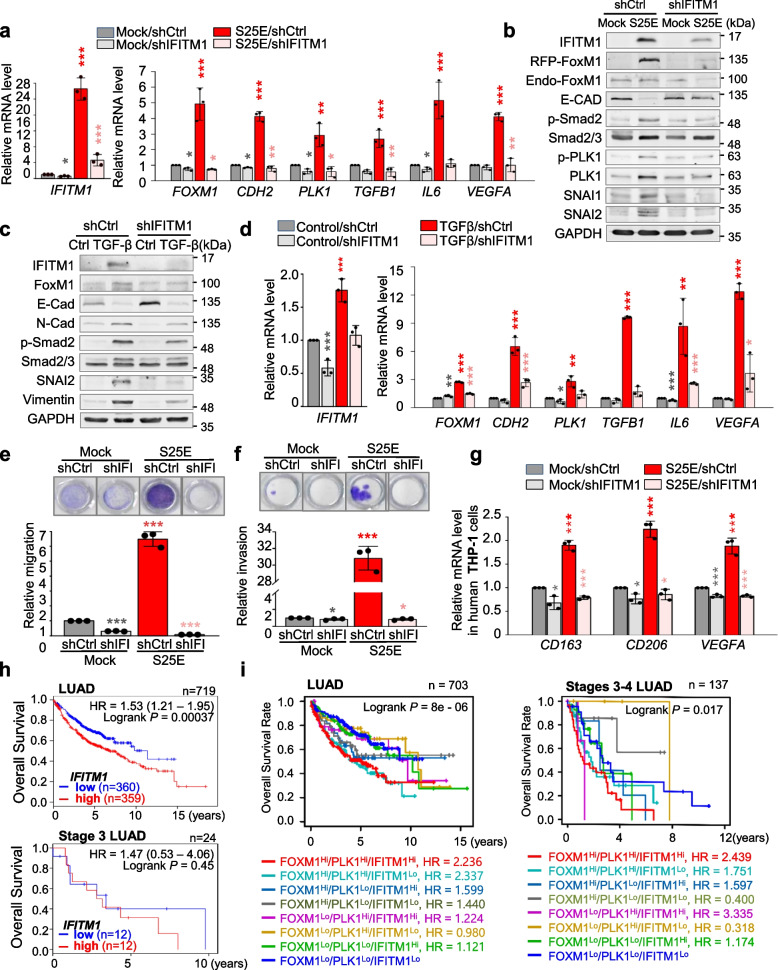
IFITM1 functions as a regulator of metastasis and polarization of M2d-TAM in p-FoxM1-induced metastasis. **a**, **b** IFITM1 was depleted in A549^S25E^ cells using human IFITM1 shRNA for 48 h. **a** qRT-PCR was performed for *IFITM1*, *FOXM1*, *CDH2*, *PLK1*, *TGFB1*, *IL6*, and *VEGFA*. **b** Immunoblot analyses were performed using anti-IFITM1, anti-RFP, anti-E-cadherin, anti-p-Smad2, anti-Smad2/3, anti-SNAI1, anti-SNAI2, and anti-GAPDH antibodies. **c**, **d** 5 ng/mL TGF-β was applied to A549 cells depleting IFITM1 with shRNA. **c** Immunoblotting was performed using anti-IFITM1, anti-FoxM1, anti-E-cadherin, anti-N-cadherin, anti-p-Smad2, anti-Smad2/3, anti-SNAI2, anti-vimentin, and anti-GAPDH antibodies. **d** qRT-PCR was performed for *IFITM1*, *FOXM1*, *CDH2*, *PLK1*, *TGFB1*, *IL6*, and *VEGFA*. **e** IFITM1 was depleted in A549^S25E^ cells using human IFITM1 shRNA for 48 h. Cells were subjected to a Transwell migration assay. Three days after seeding, the cells on the bottom surface were stained with 0.05% crystal violet dye. Images of the Transwell cell migration assay were collected and analyzed with an Odyssey infrared imaging system and plotted. **f** An invasion assay was performed using A549 cells expressing wild-type or mutants of FoxM1. Seven days after seeding, the cells that invaded the bottom surface were stained with 0.05% crystal violet dye, and the relative absorbance was plotted (*n* = 3). **g** THP-1 cells were co-cultured with A549^S25E^ cells depleting IFITM1 for 48 h. qRT-PCR was performed for *CD163*, *CD206*, and *VEGFA*. **h** The overall survival (OS) of all LUAD patients (*n* = 719) (**h**, upper) and stage 3 LUAD patients (*n* = 24) (**h**, lower) was analyzed according to *IFITM1* expression level using KM PLOTTER. High (Hi) and low (Lo) were generated based on the expression at the median cut-off. **i** The OS of all LUAD patients (*n* = 703) (**i**, left) and stage 3–4 LUAD patients (*n* = 137) (**i**, right) was analyzed according to *IFITM1*, *FOXM1*, and *PLK1* expression levels using KM PLOTTER. High (Hi) and low (Lo) were generated based on the expression at the median cut-off. Data are presented as mean ± SD of three independent experiments (significantly different from the experimental control). *p < 0.05; ***p < 0.001; (*n* = 3)

In A549^S25E^ cells, IFITM1 was the highest expressed factor among the top five genes (Fig. 5e-f). To determine the effect of IFITM1 on metastasis and macrophage polarization, IFITM1 was depleted with viral shRNA targeting human *IFITM1* (Fig. 8, Fig. S12a). When IFITM1 expression was downregulated in IFITM1-depleted A549^S25E^ cells, the levels of *CDH2*, *TGFB1*, *IL6*, and *VEGFA* upregulated by FoxM1^S25E^ were markedly reduced compared with the scramble control (Fig. 8a), indicating IFITM1 affects p-FoxM1^S25^-mediated EMT activation and TAM polarization. In addition, the levels of *IL6* and *VEGFA* were reduced by *IFITM1* knock-down in A549^S25E^ (A549^S25E_shIFITM1^) cells compared with those of A549^S25E^ cells treated with scramble shRNA control (A549^S25E_shControl^). Immunoblot analysis revealed that levels of IFITM1, FoxM1, p-Smad2, and SNAI1 were lower in A549^S25E_shIFITM1^ than A549^S25E_shControl^ (Fig. 8b). Notably, p-PLK1^T210^ level was also downregulated in A549^S25E_shIFITM1^ compared with that of A549^S25E_shControl^. FoxM1 or IFITM1 depletion mutually reduced each level (Fig. 8a, Fig. S12b), while exogenous expression of FoxM1 or IFITM1 mutually upregulated each other in A549 cells (Fig. 5, Fig. S12c-d), suggesting that IFITM1 and FoxM1 closely but not directly regulate mutual expression (Fig. 7i). When *IFITM1* was depleted during TGF-β-induced EMT, the upregulation of N-cadherin, vimentin, SNAI2, and p-Smad2 by TGF-β treatment was reduced by IFITM1 knock-down (Fig. 8c). Consistent with Fig. 8a, IFITM1 depletion suppressed the expression of *CDH2*, *TGFB1*, *IL6*, and *VEGFA* more strongly than that of control shRNA in TGF-β-induced EMT (Fig. 8d). Because IFITM1 depletion reduced the expression of mesenchymal markers and *TGFB1* (Fig. 8a-d), the motility and invasiveness were determined using Transwell and/or the invasion system. Motility and invasiveness in A549^S25E_shIFITM1^ were reduced approximately 6- and 30-fold, respectively, compared with A549^S25E_shControl^ cells (Fig. 8e-f), indicating that IFITM1 is important in the progression of cell migration and invasion. To further investigate whether knock-down of IFITM1 can affect M2-like TAM polarization, M2d markers *CD163*, *CD206*, and *VEGFA* were observed in THP-1 cells co-cultured with A549^S25E_shIFITM1^ (Fig. 8g). The M2d markers were more markedly reduced in THP-1 cells co-cultivated with A549^S25E_shIFITM1^ cells than in A549^S25E_shControl^ cells (Fig. 8g), indicating that IFITM1 is an important trigger of M2d-TAM polarization. Therefore, IFITM1 upregulated by p-FoxM1^S25^ should be important to regulate cancer metastasis and TAM polarization in the TME.

### Clinical relevance of PLK1, IFITM1, and FOXM1 in advanced LUAD

We next investigated the clinical relevance between *IFITM1* expression and survival rates of advanced LUAD in patients. The analysis (Fig. 8h, Table S9) showed that IFITM1 expression is significantly correlated with OS of LUAD patients (Fig. 8h, upper panel; *n* = 719, HR = 1.53, log rank *P* = 0.00037) but not that of LUSQ patients (Fig. S12e; *n* = 524, HR = 0.92, log rank *P* = n.s.). Additional analysis between OS and co-expression of *IFITM1*, *FOXM1*, and *PLK1* in LUAD patients showed that the OS with high *FOXM1/PLK1/IFITM1* expression was significantly shorter than that with low *FOXM1/PLK1/IFITM1* expression (*n* = 703, log-rank *P* = 8e-06) (Fig. 8i, left panel). Furthermore, in a clinical analysis of advanced LUAD patients at stages 3–4, the OS of patients with high *FOXM1/PLK1/IFITM1* expression was shorter than for those with low *FOXM1/PLK1/IFITM1* (*n* = 137, log-rank *P* = 0.017) (Fig. 8i, right panel). The cumulative OS in LUAD patients revealed that high expression of at least two of *PLK1*, *IFITM1*, and *FOXM1* highly correlated with OS of advanced LUAD patients as well as all stages of LUAD (Fig. 8i). To understand *IFITM1* expression in metastatic LUAD, a heatmap was analyzed based on the degree of expression of *IFITM1* from TCGA data analysis in normal and tumor tissue, depending on stage of LUAD (Fig. S12f). Concurrent expression of *IFITM1*, *FOXM1*, and *PLK1* showed a high correlation with tumor formation in primary and advanced LUAD but not in LUSQ. Therefore, additional expression of *IFITM1* with *FOXM1* and *PLK1* should affect the survival rates of advanced LUAD.

## Discussion

**Fig. 9:**
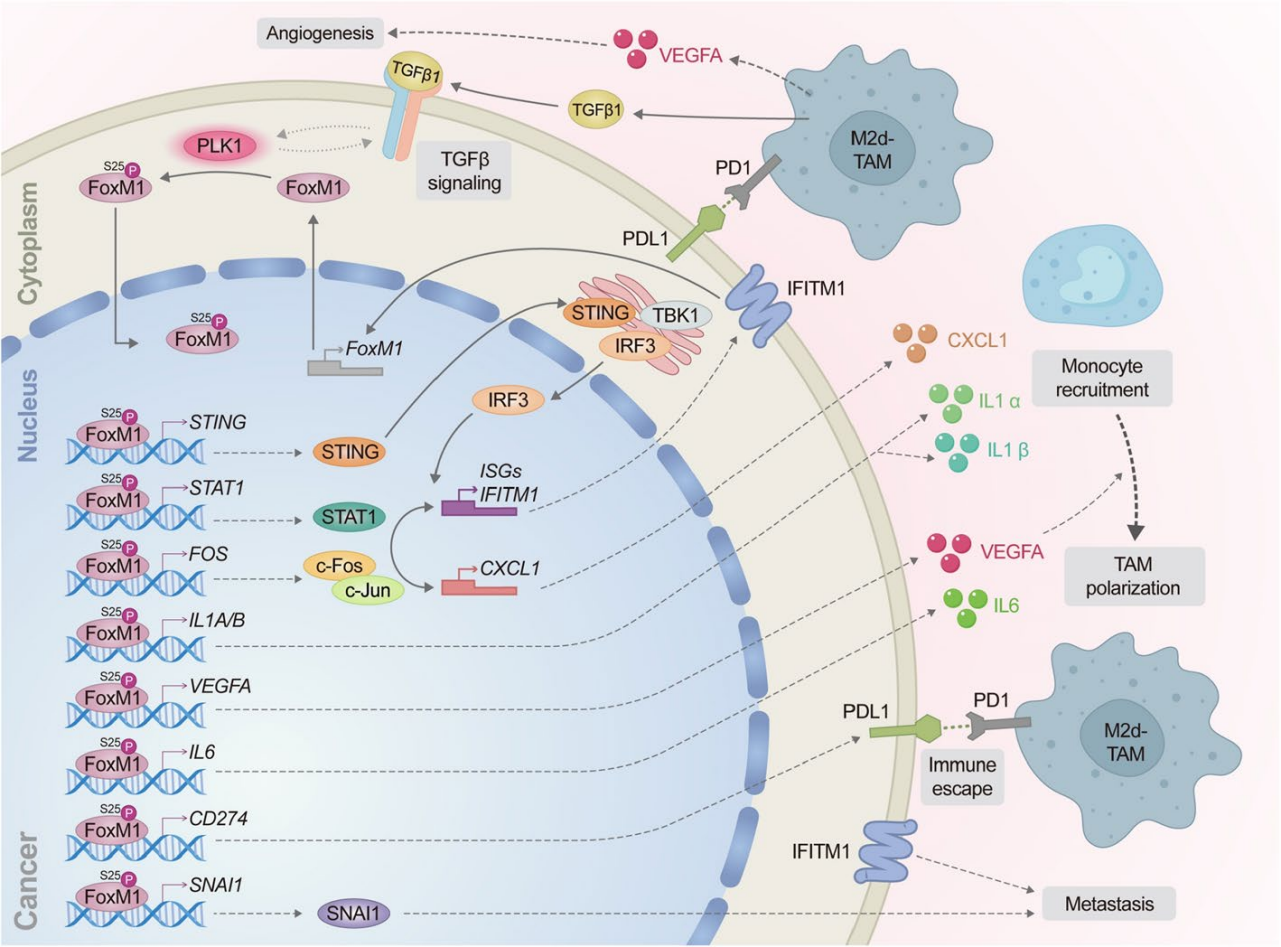
A plausible model of p-FoxM1^S25^-based monocyte recruitment, TAM polarization, angiogenesis, immune escape, and metastasis. In the cytoplasm, PLK1 phosphorylates FoxM1 at S25 in TGF-β-induced EMT, which triggers the nuclear translocation of p-FoxM1. p-FoxM1^S25^ directly activates *STING*, *FOS*, *STAT1*, *IL1A*, *IL1B*, *IL6*, *VEGFA*, *CD274*, and *SNAI1* by direct binding to their promoters in the nucleus. Additionally, activated STAT1 and AP-1 (A complex of c-Fos/c-Jun) signaling facilitates the expression of *IFITM1*, *CXCL1*, *IL1A*, *IL1B*, *IL6*, *VEGFA*, *CD274*, and *SNAI1*. Upregulated IL1A, IL1B, CXCL1, and VEGFA trigger the recruitment of monocytes. IL6 induces TAM polarization. IFITM1 amplifies signaling through the upregulation of FoxM1. TGF-β and VEGFA secreted by TAM strengthen TGF-β-induced EMT of LUAD and angiogenesis in the TME, respectively. Expressed PD-L1 in LUAD escapes the immune checkpoint by binding with PD1 of TAM. Upregulated SNAI1 regulates the EMT and metastasis in LUAD

The main findings in this study are as follows. First, high expression of FoxM1, PLK1, and IFITM1 was inversely correlated with shorter OS of patients with advanced NSCLC, especially LUAD, which may be useful for prognostics. Second, phosphorylation of FoxM1 at Ser25 by PLK1 acquires the invasiveness, which enhances metastatic lung nodule formation through direct activation of the EMT and immune escape in an in vivo mouse model. Third, p-FoxM1^S25^ triggers the expression of cytokines and chemokines via AP-1 (c-Fos/c-Jun), NF-κB, and STAT1 signaling, inducing monocyte recruitment near LUAD and triggering polarization into M2d-TAMs in the TME (Fig. 9). Subsequently, M2d-TAMs accelerate malignancy through metastasis and angiogenesis by upregulating *TGFB1* and *VEGFA* (Fig. 9). Notably, *IL6* was upregulated in A549^S25E^ cells, functioning as an inducer for macrophage polarization into M2d-TAMs [48]. Fourth, PD-L1 expression in metastatic lung tumors of mice having A549^S25E^ cells promoted immune checkpoint evasion in the TME. Fifth, IFITM1 is the most highly expressed gene in invasive A549^S25E^ cells through activation of STING/TBK1/IRF3, JAK1/STAT1, and/or AP-1 signaling but not NF-κB signaling. The invasiveness and TAM polarization induced by p-FoxM1^S25^ are reduced by IFITM1 depletion accompanying the reduction of FoxM1. Thus, IFITM1 and FoxM1 closely, but not directly, regulate mutual expression, demonstrated by experiments of gain-of-function or loss-of-function and ChIP analysis. Therefore, invasive FoxM1 phosphorylated at Ser25 by PLK1 translocates into the nucleus and triggers the expression of genes related to TAM polarization, angiogenesis, metastasis, and immune escape, which are amplified by IFITM1, reinforcing the malignant characteristics of LUAD in the TME (Fig. 9).

FoxM1, a putative EMT regulator and a determinant between mitogenic and invasive phenotypes of cancer [56], affects migration of macrophages [46, 47] and activation of lung fibroblasts [57] in the local TME. Catalytically active PLK1, a kinase of FoxM1 in mitosis [28], drives the EMT and promotes tumor survival through activation of TGF-β signaling [29] and PD-L1 expression [30]. Phosphorylation of FoxM1 at Ser25 by PLK1 is critical for acquiring the invasiveness to regulate the EMT and crosstalk with microenvironmental cells. However, its phosphorylation at Ser715 by PLK1 after priming phosphorylation at Thr596 by Cdk1 facilitates mitotic cell proliferation [28], consistent with the proliferation assay (Fig. 3c). In addition, its phosphorylation at Ser361 is related to DNA repair, based on the report of Chk2-mediated phosphorylation of FoxM1 at Ser361 in response to DNA damage for stimulating DNA repair genes [58]. This agreed with our findings that phosphorylation at Ser361 or Ser715 did not affect the mesenchymal transition or cell migration, except for Ser25 in LUAD. Therefore, phosphorylation of FoxM1 at Ser25 by PLK1 should be specified for cancer metastasis and direct activation of mesenchymal genes to acquire invasiveness.

The main biological processes of non-invasive cells expressing FoxM1^S25E^ were related to cell migration, adhesion, and blood circulation in the GO analysis (Fig. 5), while the main processes of invasive cells expressing FoxM1^S25E^ were related to stress response, interferon signaling activation, and cytokine response. The effects producing pro-inflammatory cytokines and ISGs were remarkable when cells were expressed with FoxM1^S25E^. LUAD having p-FoxM1^S25^ can recruit macrophages to the TME and polarize M2d-TAM, as demonstrated by immunohistochemistry with CD68- and CD163-positive lung tissues of mice. Additionally, M2d-TAMs co-cultured with A549^S25E^ cells released TGF-β1 and VEGFA, amplifying the EMT of A549^S25E^ cells. Polarization of macrophages to TAMs for immune escape is important for solid tumor survival because PD-1 expression in macrophages regulates tumor immunity and phagocytic potency against tumor cells [52]. The expression of PD-L1 in A549^S25E^ was regulated by direct activation by FoxM1^S25E^ as well as several pathways including STAT1, AP-1, and NF-κB signaling, whereas the expression of IFITM1 in A549^S25E^ was regulated by STAT1 and AP-1 signaling but not by FoxM1^S25E^. IFITM1 expression was positively related to tumor proliferation, invasion, and metastasis [59–62]. Previously, IFN-γ was shown to upregulate de novo IFITM1 synthesis through stimulation of the interferon-stimulated response element of its promoter region [63]. In STAT1-defective U3A cells, IFITM1 induction was eliminated in response to IFN-γ and was recovered by STAT1 expression [64], which supported our data that STAT1 signaling upregulates the expression of IFITM1 in invasive LUAD^S25E^ cells. Direct expression of STAT1 and c-Fos by p-FoxM1^S25^ regulates IFITM1 expression with the highest level in metastatic LUAD, which amplifies FoxM1 signaling.

Although the new findings concerning the role of phosphorylated FoxM1 in invasive LUAD are noteworthy in this study, there remains a need to conduct further research into the immune components activated by p-FoxM1 within patients’ systems.

## Conclusion

Our findings provide that FoxM1 drives the invasiveness by PLK1-mediated phosphorylation at Ser25 through triggering the expression of genes for TAM polarization, immune escape, and metastasis, functioning as a direct transcriptional factor for reinforcing LUAD malignancy in the TME. Invasive FoxM1 regulates IFITM1 expression by activating of STING/TBK1/IRF3, JAK1/STAT1, and/or AP-1 signaling to amplify FoxM1-mediated signaling. Therefore, inhibitory strategies for invasive FoxM1 based on its degradation, dephosphorylation, or inhibition of its transcription activity can be useful to suppress cancer metastasis and immune checkpoint evasion of cancer cells. These data reveal a novel reprogramming function of FoxM1 acquired by phosphorylation in TME and identify potential therapeutic strategies for LUAD.

### Supplementary Information

**Additional file 1.**

## Data Availability

Source data and the microarray data generated in this study are provided with this paper. Previously published RNAseq and microarray data that were reanalysed here were derived from the websites of Morpheus (https://software.broadinstitute.org/morpheus), cBioPortal (https://www.cbioportal.org/), KM PLOTTER (https://kmplot.com/analysis/), and the TCGA Research Network (https://portal.gdc.cancer.gov/). The authors declare that all the data supporting the findings of this study are available within the paper and its Supplementary Information files. All other data supporting the findings of this study are available from the corresponding author upon reasonable request.

## Abbreviations

A549^WT^: A549 cells expressing wild-type FoxM1
A549^S25A^: A549 cells expressing non-phosphomimetic alanine substitute of FoxM1 at Ser25
A549^S25E^: A549 cells expressing phosphomimetic glutamate substitute of FoxM1 at Ser25
CAN: Acetonitrile
DTT: Dithiothreitol
EGTA: Egtazic acid
ELISA: Enzyme-linked immunosorbent assay
EMT: Epithelial-mesenchymal transition
FoxM1: Forehead box M1
FoxM1^S25E^: Phosphomimetic glutamate substitute of FoxM1 at Ser25
FoxM1^S25A^: Non-phosphomimetic alanine substitute of FoxM1 at Ser25
GO: Gene ontology
HR: Hazard ratio
IFI44L: Interferon-induced protein 44 like
IFITM1: Interferon-induced transmembrane protein 1
IL: Interleukin
IRF3: Interferon regulatory factor 3
ISGs: Interferon-stimulated genes
KM plot: Kaplan Meier plot
LC: Liquid chromatography
LUAD: Lung adenocarcinoma
LUSQ: Lung squamous cell cancer
MS: Mass spectrometry
MX1: MX dynamin-like GTPase 1
NSCLC: Non-small cell lung cancer
OS: Overall survival
RFP: Red fluorescent protein
PD-1: Programmed cell death protein -1
PD-L1: Programmed cell death-ligand 1
PLK1: Polo-like kinase 1
RT: Room temperature
SDS-PAGE: Sodium dodecyl sulfate—polyacrylamide gel electrophoresis
SNAI1: Snail family transcriptional repressor 1
STAT1: Signal transducer and activator of transcription 1
STING: Stimulator of interferon genes
TAMs: Tumor-associated macrophages
TBK1: Tank binding kinase 1
TLR: Toll-like receptor
TCGA: The Cancer Genome Atlas
TCTP: The translational controlled tumour protein
TGF-β: Transforming growth factor-β
TME: Tumor microenvironment
VEGFA: Vascular endothelial growth factor A
WT: Wild-type
XAF1: XIAP Associated Factor 1
