# Supplementary material for: Invasive FoxM1 phosphorylated by PLK1 induces the polarization of tumor-associated macrophages to promote immune escape and metastasis, amplified by IFITM1
Source: J Exp Clin Cancer Res. 2023 Nov 16;42:302. doi: 10.1186/s13046-023-02872-1 (PMC10652615; doi:10.1186/s13046-023-02872-1)
Supplement: Supplementary file 1 — Additional file 1. [file 13046_2023_2872_MOESM1_ESM.docx]

**Supplementary Information**

**Invasive FoxM1 phosphorylated by PLK1 induces the polarization of tumor-associated macrophages to promote immune escape and metastasis, amplified by IFITM1**

Rong Xu^1^, Young-Joo Lee^1^, Chang-Hyeon Kim^1^, Ga-Hong Min^1^, Jung-Won Park^1^, Yeo-Bin Kim^1^, Dae-Hoon Kim^1^, Jung-Hyun Kim^2^, and Hyungshin Yim^1,*^

^1^Department of Pharmacy, College of Pharmacy, **Institute of Pharmaceutical Science and Technology,** Hanyang University, Ansan, Gyeonggi-do 15588, Republic of Korea

^2^Division of Intractable Diseases Research, Department of Chronic Diseases Convergence Research, Korea National Institute of Health, Cheongju, Chungcheongbuk-do 28160, Republic of Korea

^*^Corresponding author

Hyungshin YIM

Address: Department of Pharmacy, College of Pharmacy, **Institute of Pharmaceutical Science and Technology,** Hanyang University, Ansan, Gyeonggi-do 15588, Republic of Korea

Phone: +82-31-400-5810

FAX: +82-31-400-5958

E-mail: [hsyim@hanyang.ac.kr](mailto:hsyim@hanyang.ac.kr)


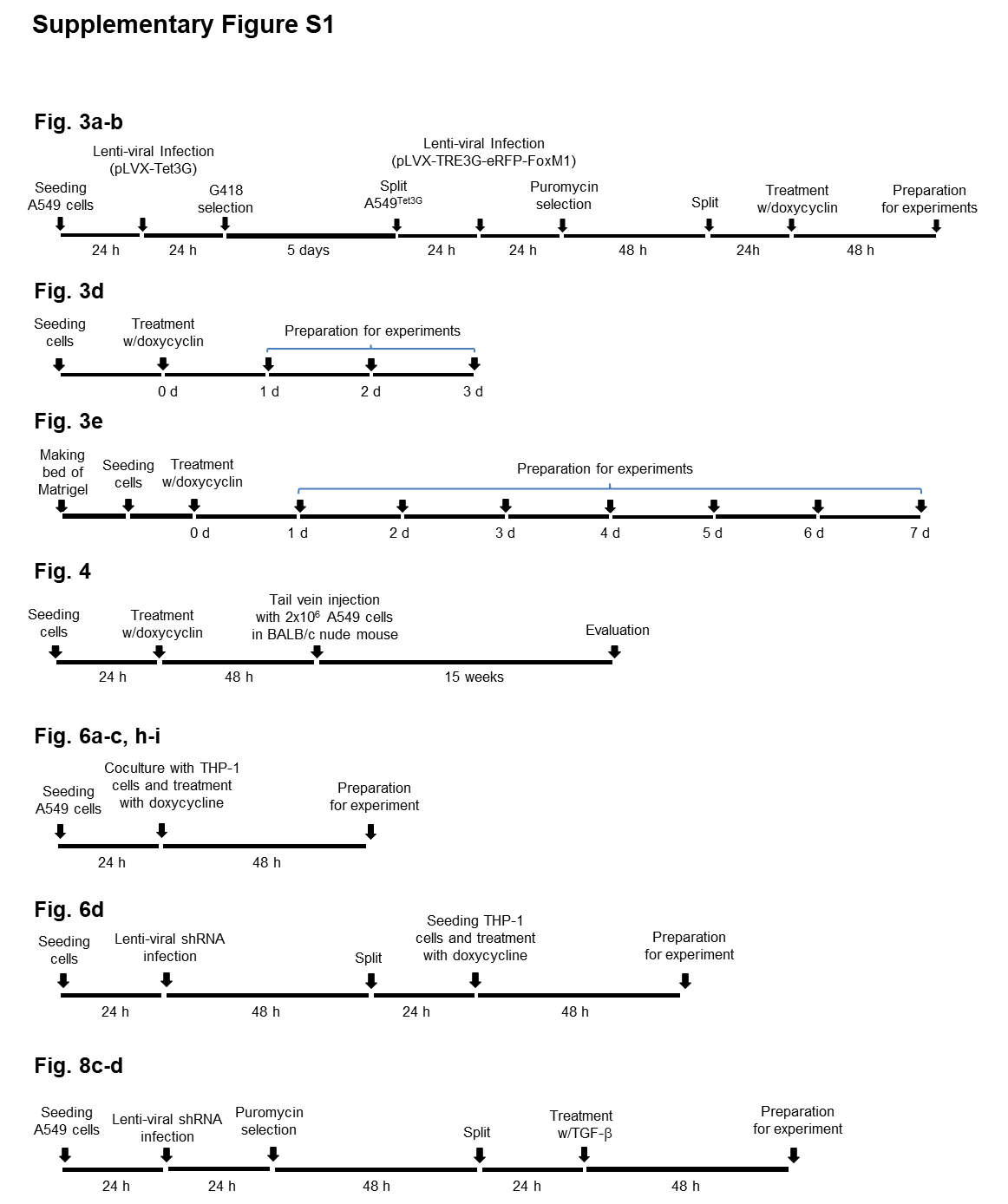


**Supplementary Figure S1.** **Schemes of experiments.**

**
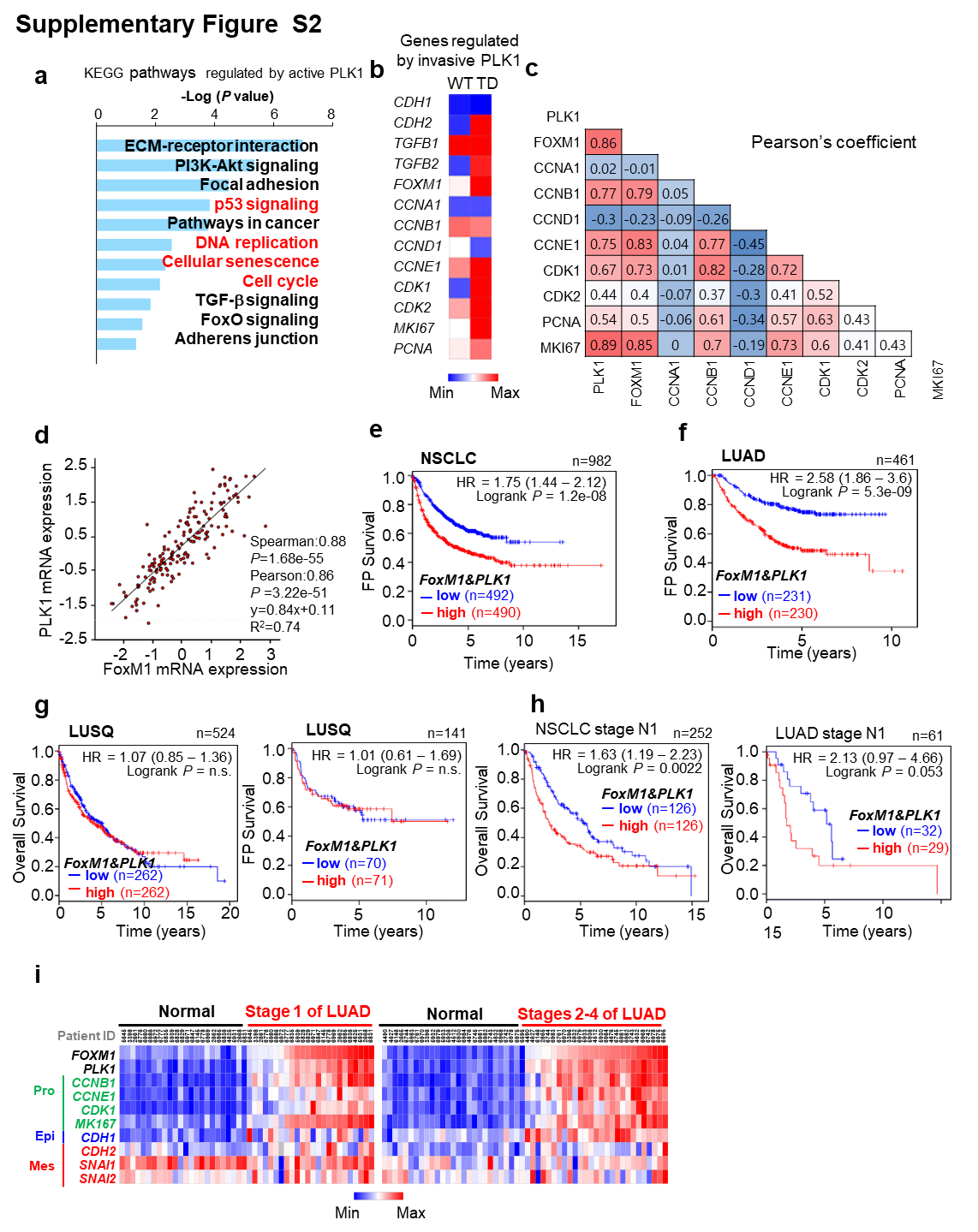
**

**Supplementary Figure S2. Concurrent upregulation of FoxM1 and PLK1 is correlated with poor survival of LUAD patients. a,** Analysis of KEGG pathways regulated by active PLK1 extracted from the microarray data^1^. **b,** A heatmap analysis was performed from cell cycle-regulatory genes regulated by invasive PLK1 using the microarray data^1^. **c,** Analysis of Pearson’s coefficient for the correlations between cell cycle-regulatory factors *PLK1*, *FOXM1*, *CCNA1*, *CCNB1*, *CCND1*, *CCNE1*, *CDK1*, *CDK2*, *PCNA*, and *MKI67* in non-small cell lung cancer (NSCLC) patients using cBioPortal. **d,** Analysis of Spearman’s and Person’s coefficients for correlations between *PLK1* and *FOXM1* in NSCLC patients using cBioPortal. **e-f,** Survival until first progression (FPS) rates of patients with NSCLC or LUAD were correlated with the levels of *FOXM1* and *PLK1* expression. FPS times of patients with NSCLC (*n =* 982) (**e**) and LUAD (*n =* 461) (**f**) were analyzed according to *PLK1* and *FOXM1* expression levels. High (Hi) *vs* low (Lo) expression was split by median cut-off value using KM plot database^2^. **g,** Overall survival rates (left) and FPS times (right) of patients with lung squamous cell carcinoma (LUSQ) were analyzed according to *PLK1* and *FOXM1* expression. High (Hi) *vs* low (Lo) expression was split by median cut-off value using KM plot database^2^. **h,** Overall survival rates of patients with TNM stage N1 NSCLC (left) and LUAD (right) were analyzed according to *PLK1* and *FOXM1* expression. High (Hi) *vs* low (Lo) expression was split by median cut-off value using KM plot database^2^. **i,** A heatmap analysis was performed from a LUAD patient dataset of TCGA for *FOXM1*, *PLK1*, proliferating markers (Pro), epithelial marker (Epi), and mesenchymal markers (Mes) in paired normal and tumor tissues at stage 1 (left) or stages 2–4 (right).

**
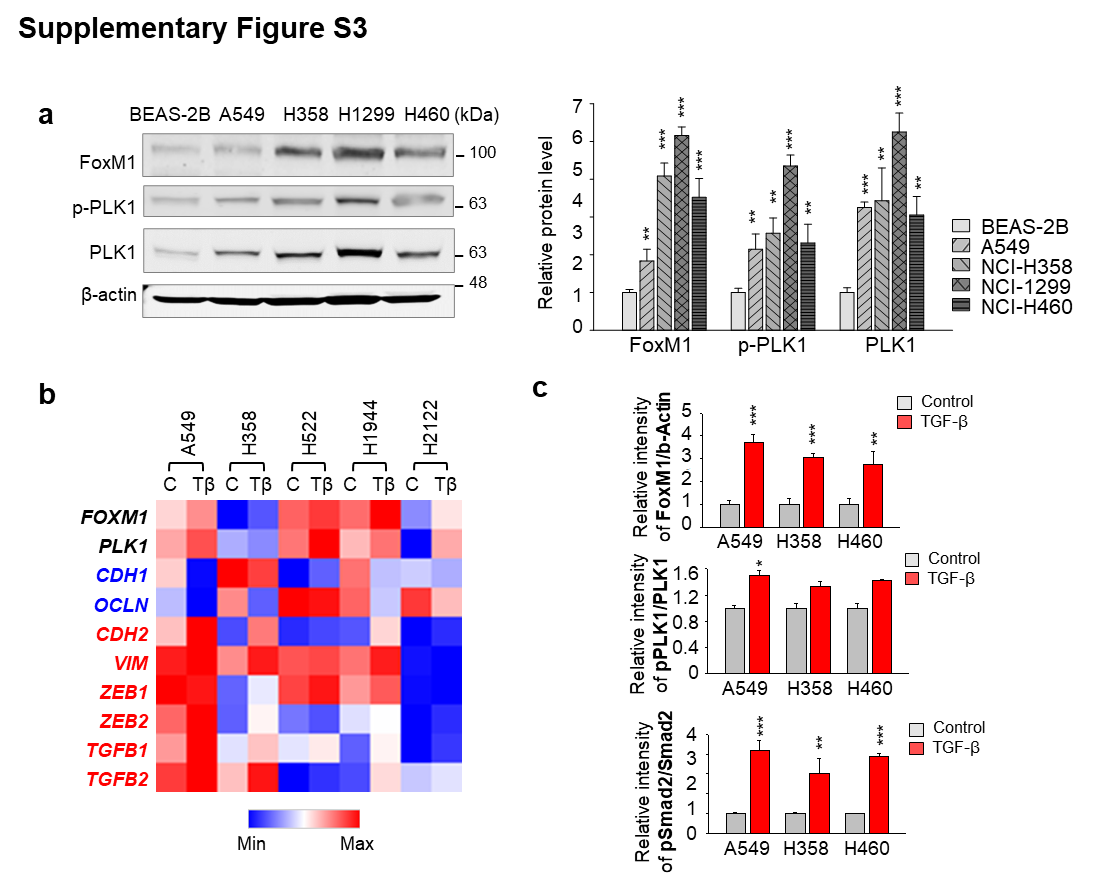
**

**Supplementary Figure S3. Concurrent upregulation of FoxM1 and PLK1 in TGF-β-induced EMT. a,** Immunoblotting was performed to measure the expression and phosphorylation of PLK1 using specific antibodies for FoxM1, PLK1, and p-PLK1 (T210) in BEAS-2B, A549, NCI-H358 (H358), NCI-H1299 (H1299), and NCI-H460 (H460) cells (left panel). The relative band intensities for FoxM1, p-T210-PLK1, and PLK1 were quantified using LI-COR Odyssey software (right panel). **b,** A heatmap analysis was performed from EMT-associated genes regulated by invasive PLK1. **c,** The relative band intensities of **Fig. 1f** for FoxM1/β-actin, p-T210-PLK1/PLK1, and p-Smad2 (S465/S467)/Smad2 were quantified using LI-COR Odyssey software.

**
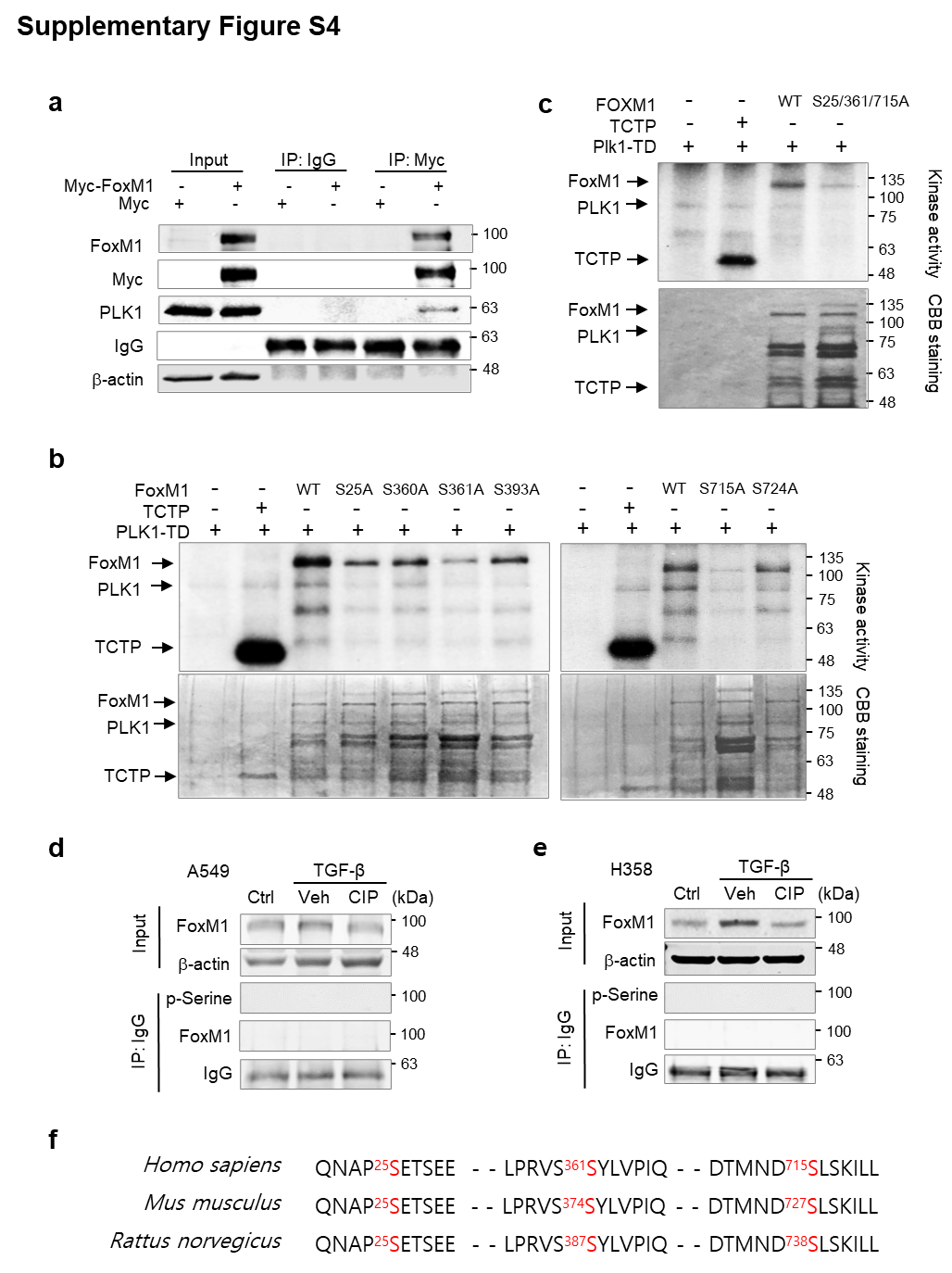
**

**Supplementary Figure S4. TGF-β-induced EMT resulted in phosphorylation of FoxM1 by PLK1 by direct interaction. a**, Myc-tagged FoxM1 was expressed in A549 cells. Immunoprecipitation of cell lysates was performed with normal IgG or anti-Myc antibodies and immunoblotting was done with anti-FoxM1, anti-Myc, and anti-PLK1 antibodies. **b,** An *in vitro* kinase assay was performed with active PLK1 (PLK1-TD), radioactive ATP, and purified GST-tagged FoxM1. GST-tagged FoxM1 was purified by site-directed single mutagenesis that substituted with alanine at S25, S360, S361, S393, S715, and S724 residues. GST-tagged TCTP was used as the positive control. **c,** An *in vitro* PLK1 kinase assay was performed with GST-tagged triple alanine mutant of FoxM1 at S25/S361/S715A (AAA) using radioactive ATP. **d**-**e**, Phosphorylation of FoxM1 occurred in A549 **(d)** and NCI-H358 **(e)** cells treated with TGF-β for 48 h. Treatment with calf intestinal alkaline phosphatase reduced the phosphorylation of FoxM1 in TGF-β-induced EMT. Immunoprecipitation of cell lysates was performed with anti-normal IgG or anti-FoxM1 (**Fig. 2f**-**g**) antibody and immunoblotting was done with anti-p-Serine antibodies. **f,** The possible residues on FoxM1 phosphorylated by PLK1 at the S25, S361, and S715 sites were evolutionarily conserved in several species including *Homo sapiens*, *Mus musculus*, *and Rattus norvegicus*.


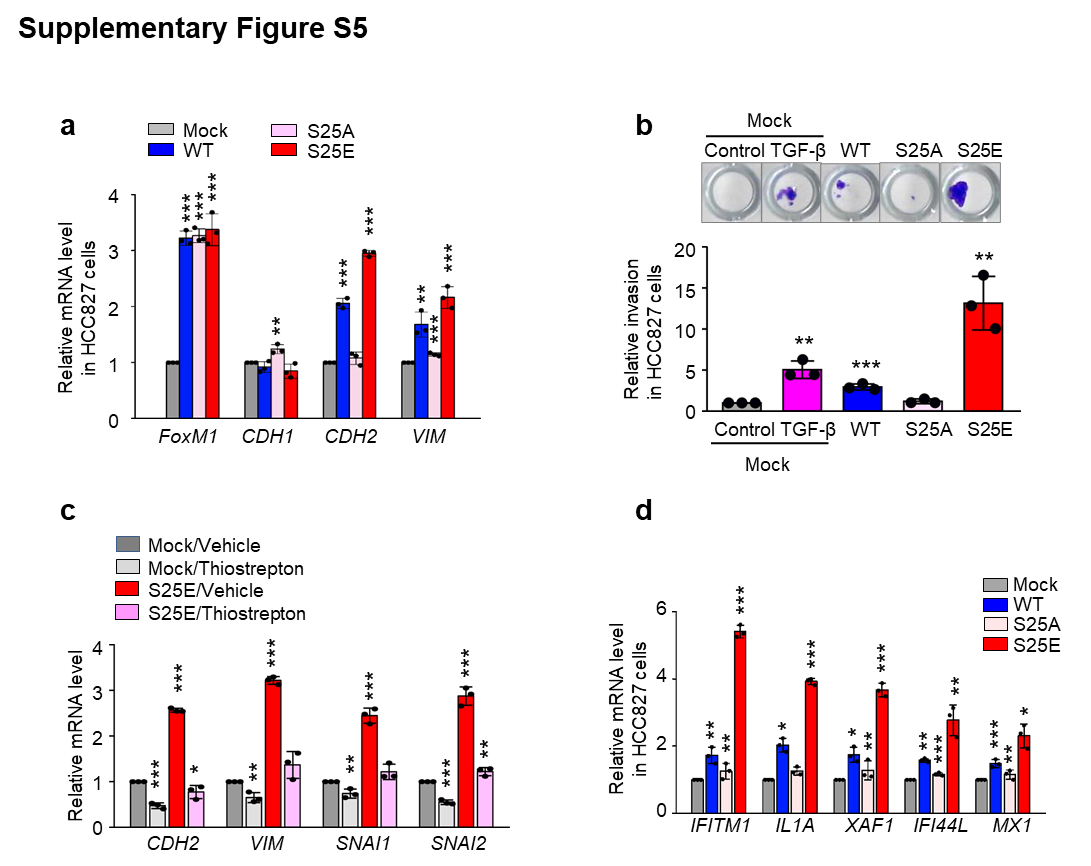


**Supplementary Figure S5. Phosphorylation of FoxM1 at Ser25 facilitates the expression of mesenchymal genes, which are reduced by treatment with the FoxM1 inhibitor thiostrepton. a-b,** RFP-tagged wild-type (WT) FoxM1 and S25A and S25E mutants were expressed in HCC827 cells. HCC827 cells were treated with doxycycline to express RFP-tagged FoxM1. **a,** qRT-PCR was performed for *FOXM1*, *CDH1*, *CDH2*, and *VIM* in HCC827 cells expressing wild-type or mutants FoxM1. **p* < 0.05; ***p* < 0.01; ****p* < 0.001; (*n =* 3). Data are presented as mean ± SD. **b**, An invasion assay was performed using HCC827 cells expressing wild-type or mutants of FoxM1. Fourteen days after seeding, the cells that invaded the bottom surface were stained with 0.05% crystal violet dye, and the relative absorbance was plotted. Data are presented as mean ± SD of at least three independent experiments (significantly different from the experimental control). **p* < 0.05; ***p* < 0.01; ****p* < 0.001 compared with experimental control. **c,** A549 cells RFP-tagged WT, S25A, and S25E of FoxM1 were expressed in A549 cells. 5 μM Thiostrepton, a FoxM1 inhibitor, was applied for 48 h. qRT-PCR was performed for *CDH2*, *VIM*, *SNAI1*, and *SNAI2*. **p* < 0.05; ***p* < 0.01; ****p* < 0.001; (*n =* 3). Data are presented as mean ± SD. **d**, qRT-PCR was performed for the top five genes *IFITM1*, *XAF1*, *IF44L*, *MX1*, and *IL1A* in total HCC827 cells expressing FoxM1. **p* < 0.05; ***p* < 0.01; ****p* < 0.001; (*n =* 3). Data are presented as mean ± SD.

**
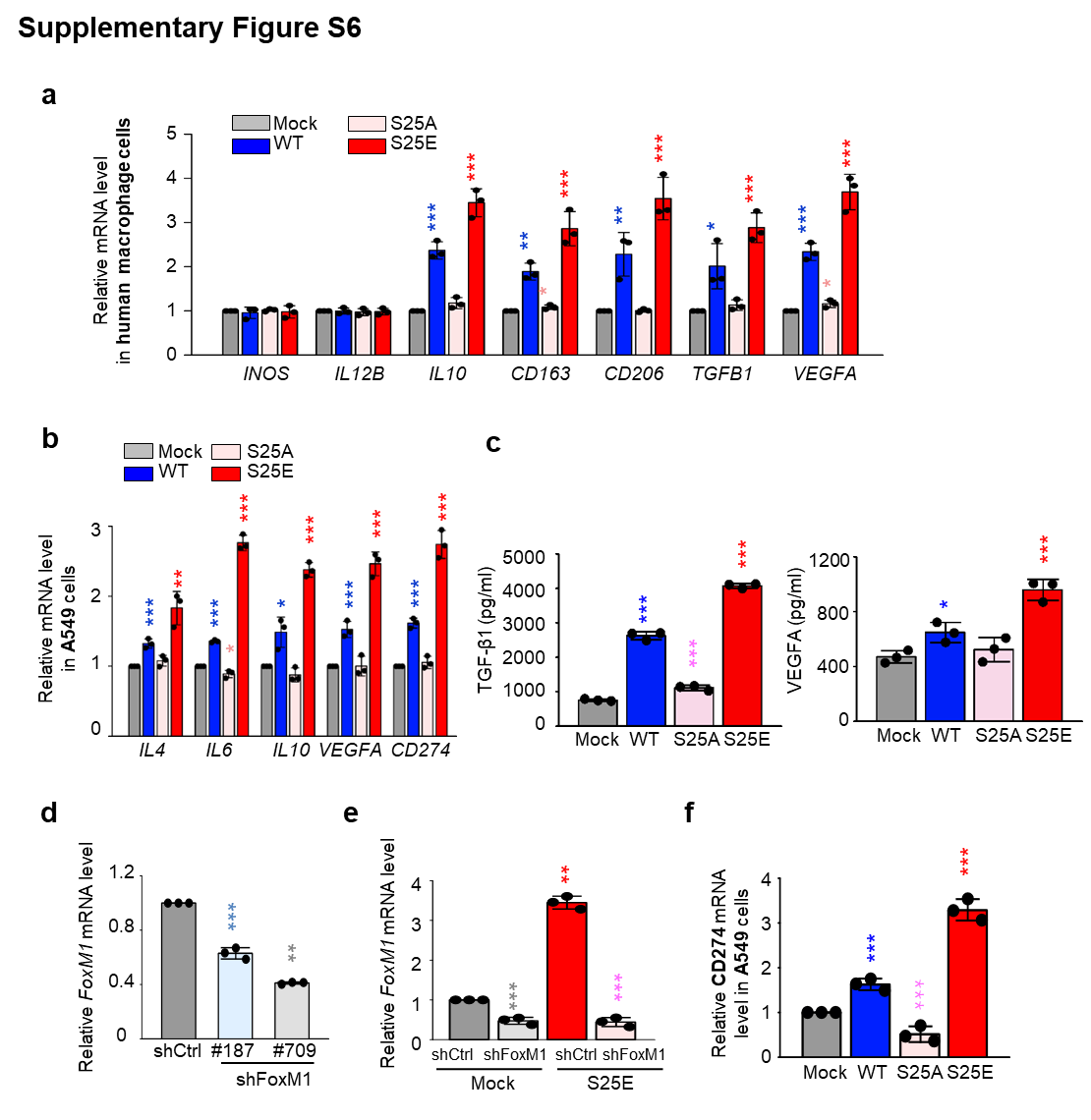
Supplementary Figure S6. p-FoxM1^S25^ functions in polarization of M2-like TAM and PD-L1 expression for cancer survival. a**, Primary human macrophages derived from human pluripotent stem cells were co-cultured with A549 cells expressing mock, wild-type (WT), S25A, and S25E FoxM1 for 48 h. qRT-PCR was performed using co-cultured primary human macrophages for markers of M1 (*INOS*, *IL12B*), M2 (*IL10*, *CD163*, *CD206*), and TAM (*TGFB1*, *VEGFA*). **b**, Primary human macrophages were co-cultured with A549 cells expressing mock, WT, S25A, and S25E FoxM1. In A549 cells, qRT-PCR was performed for *IL4*, *IL6*, *IL10*, *VEGFA*, and *CD274*. **c**, HCC827^S25E^ cells were co-cultured with human THP macrophage cells. The secreted levels of TGF-β1 and VEGFA from human THP macrophages co-cultured with A549 cells were detected using ELISA. **d**, FoxM1 shRNA targeting at the position of 187–207 or 709–729 was applied to A549 cells. qRT-PCR was performed for *FOXM1.* ***p* < 0.01; ****p* < 0.001; (*n =* 3). **e**, A549 cells expressing mock, WT, S25A, and S25E FoxM1 were treated with FoxM1 shRNA (#709) for 48 h, and qRT-PCR was performed for *FOXM1.* ***p* < 0.01; ****p* < 0.001; (*n =* 3). **f**, A549 cells expressing mock, WT, S25A, and S25E FoxM1. qRT-PCR was performed for *CD274.* (Significantly different from the experimental control). ****p* < 0.001; (*n =* 3). Data are presented as mean ± SD.

**
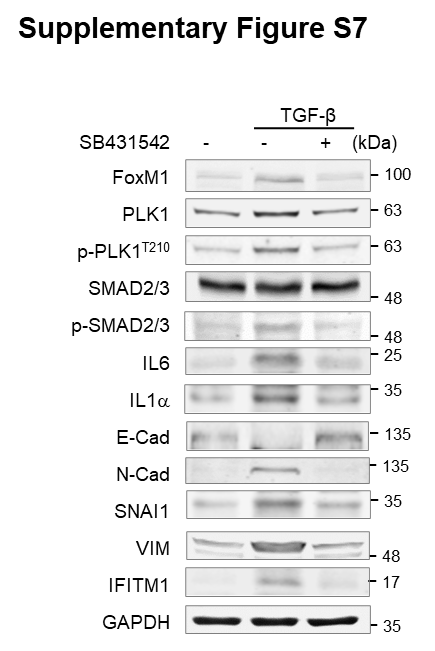
**

**Supplementary Figure S7**. TGF-β inhibitor SB431542 blocked the activation of PLK1 and levels of FoxM1, IL6, N-Cadherin, SNAI1, vimentin, and IFITM1 in A549 cells. Immunoblotting was performed using A549 cells treated with TGF- β and/or its inhibitor SB431542. FoxM1, PLK1, p-PLK1, Smad2/3, p-Smad2/3, IL6, IL β, N-cadherin, SNAI1, vimentin, IFITM1, and GAPDH were detected using specific antibodies.

**
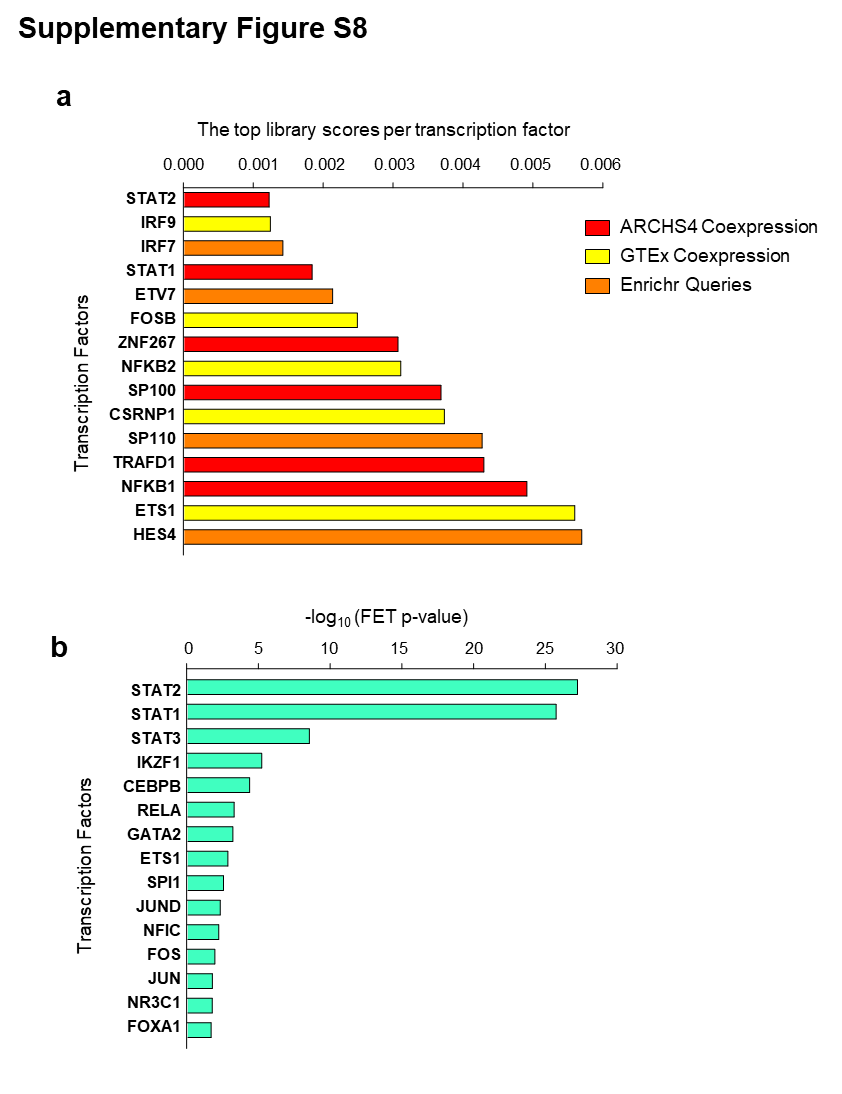
**

**Supplementary Figure S8. Prediction of transcription factors in invasive cells having phosphorylated FoxM1 at S25.** Using non-invasive and invasive A549 cells expressing S25E of FoxM1, transcriptome profiles were analyzed by microarray. The transcriptome data were clustered by gene probes with fold change >2 in cells expressing S25E FoxM1. The prediction of transcription factors for these genes was performed by Appyter database^3^. Top ranked transcription factors were displayed within 15 with 2 of library threshold. **a,** Bar chart of top ranks across all libraries. The top ranked transcription factors according to their top integrated score across all the libraries. **b,** Bar chart of scores based on FET p-values. Transcription factors ranked by the ENCODE ChIP seq gene set library.

**
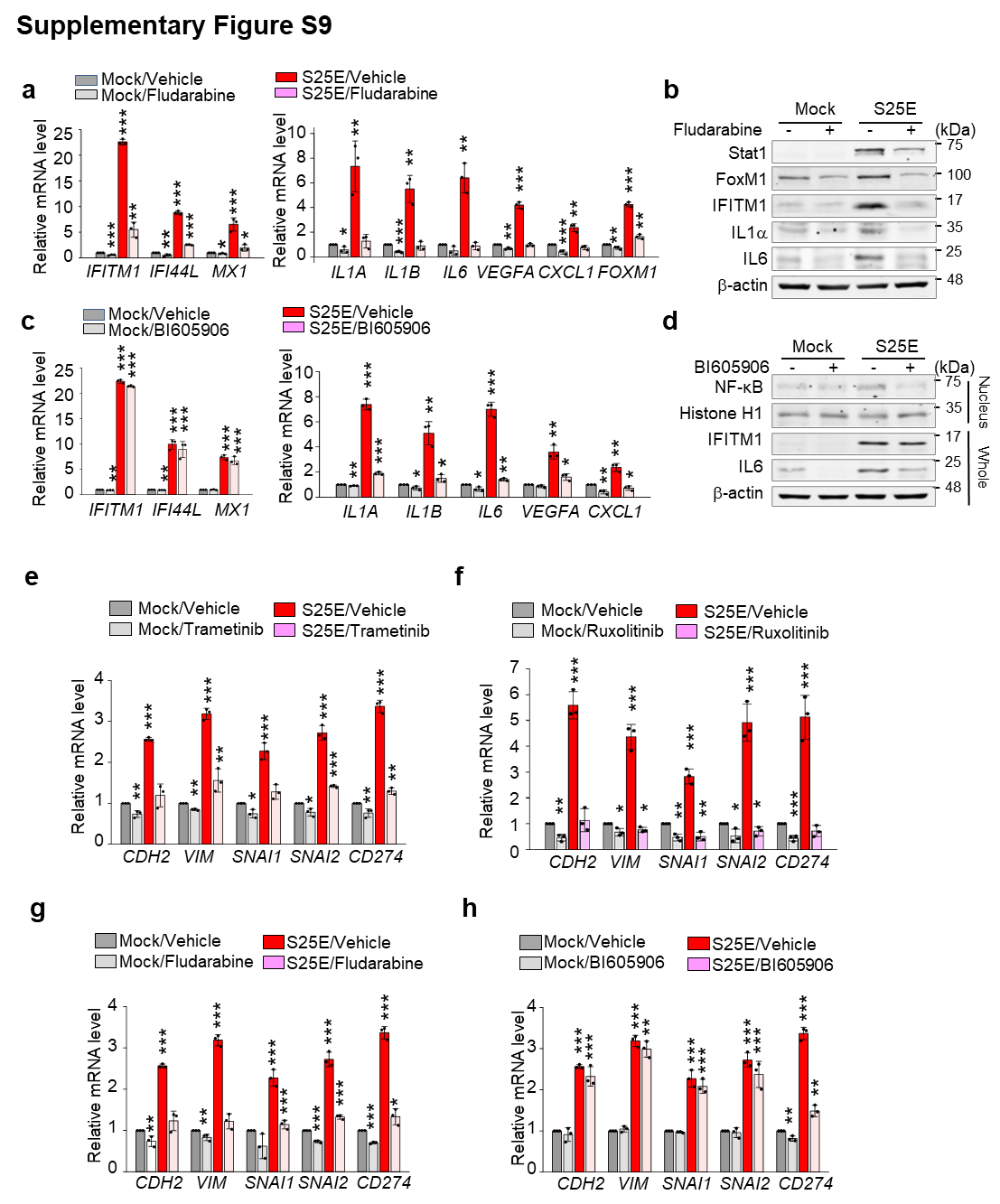
**

**Supplementary Figure S9.** **p-FoxM1^S25^ upregulates the genes related to ISGs, TAM polarization, and EMT by MEK or JAK/STAT1 signaling. a-b,** A549^S25E^ cells were treated with 1 μM fludarabin, a STAT1 inhibitor, for 48 h. **a**, qRT-PCR was performed for *IFITM1*, *IF44L*, *MX1*, *IL1A*, *IL1B*, *IL6*, *VEGFA*, *CXCL1*, and *FOXM1* in A549^S25E^ cells. **b**, Immunoblot analyses were performed using anti-FoxM1, anti-STAT1, anti-IFITM1, anti-IL1A, and anti-β-actin. **c-d,** A549^S25E^ cells were treated with 10 μM BI605906, an inhibitor of IKKβ, for 48 h. **c**, qRT-PCR was performed for *IFITM1*, *IF44L*, *MX1*, *IL1A*, *IL1B*, *IL6*, *VEGFA*, and *CXCL1* in A549^S25E^ cells. **p* < 0.05; ***p* < 0.01; ****p* < 0.001. Data are presented as mean ± SD. **d**, Immunoblotting was performed using whole and nuclear lysates of A549^S25E^ cells. Anti-NF-kB, anti-IFITM1, anti-IL1A, and anti-β-actin antibodies were used. Histone H1, a nuclear loading marker. **e-h,** A549 cells expressing S25E FoxM1 were treated with 1 μM trametinib (**e**), 15 μM ruxolitinib (**f**), 1 μM fludarabin (**g**), and 10 μM BI605906 (**h**) for 48 h. qRT-PCR was performed for *CDH2*, *VIM*, *SNAI1*, *SNAI2*, and *CD274*. **p* < 0.05; ***p* < 0.01; ****p* < 0.001. Data are presented as mean ± SD.


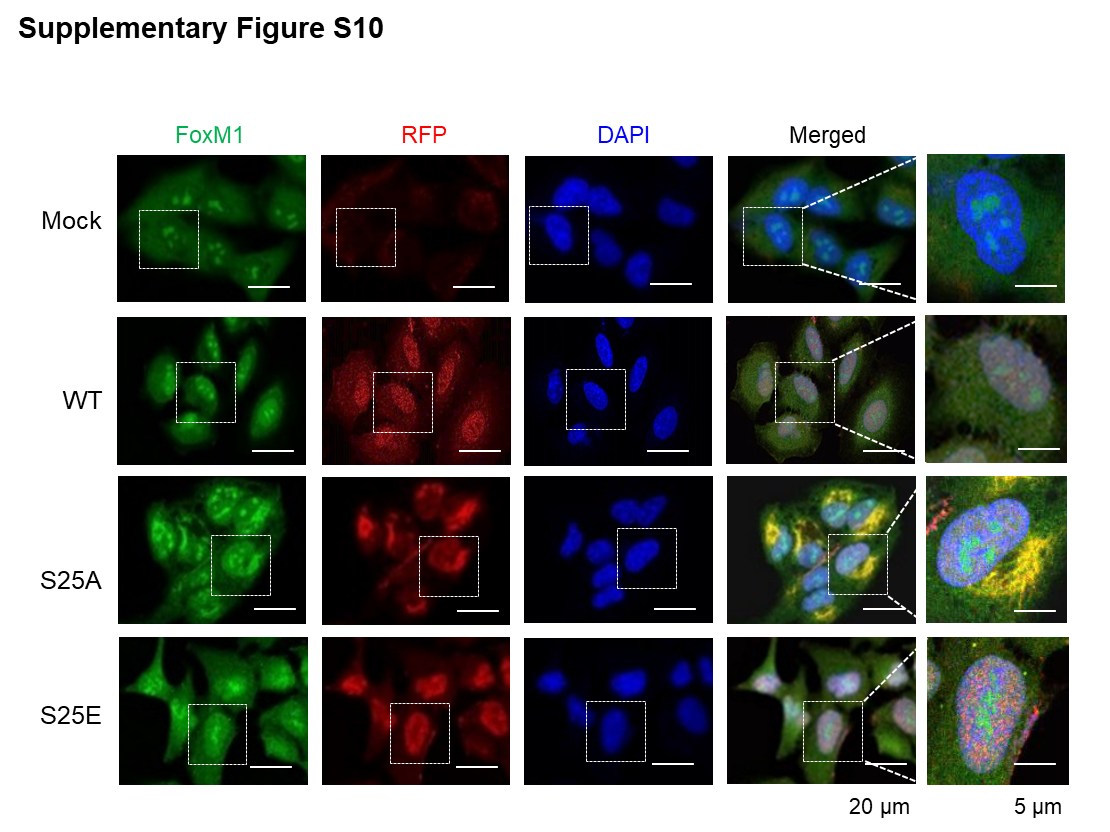


**Supplementary Figure S10. The phosphorylation of FoxM1 at S25 upregulates its nuclear translocation.** Immunofluorescence was performed with A549 cells expressing WT, S25A, or S25E mutant of FoxM1. FoxM1 (green), RFP (red), and DNA (DAPI, blue) were displayed.

**
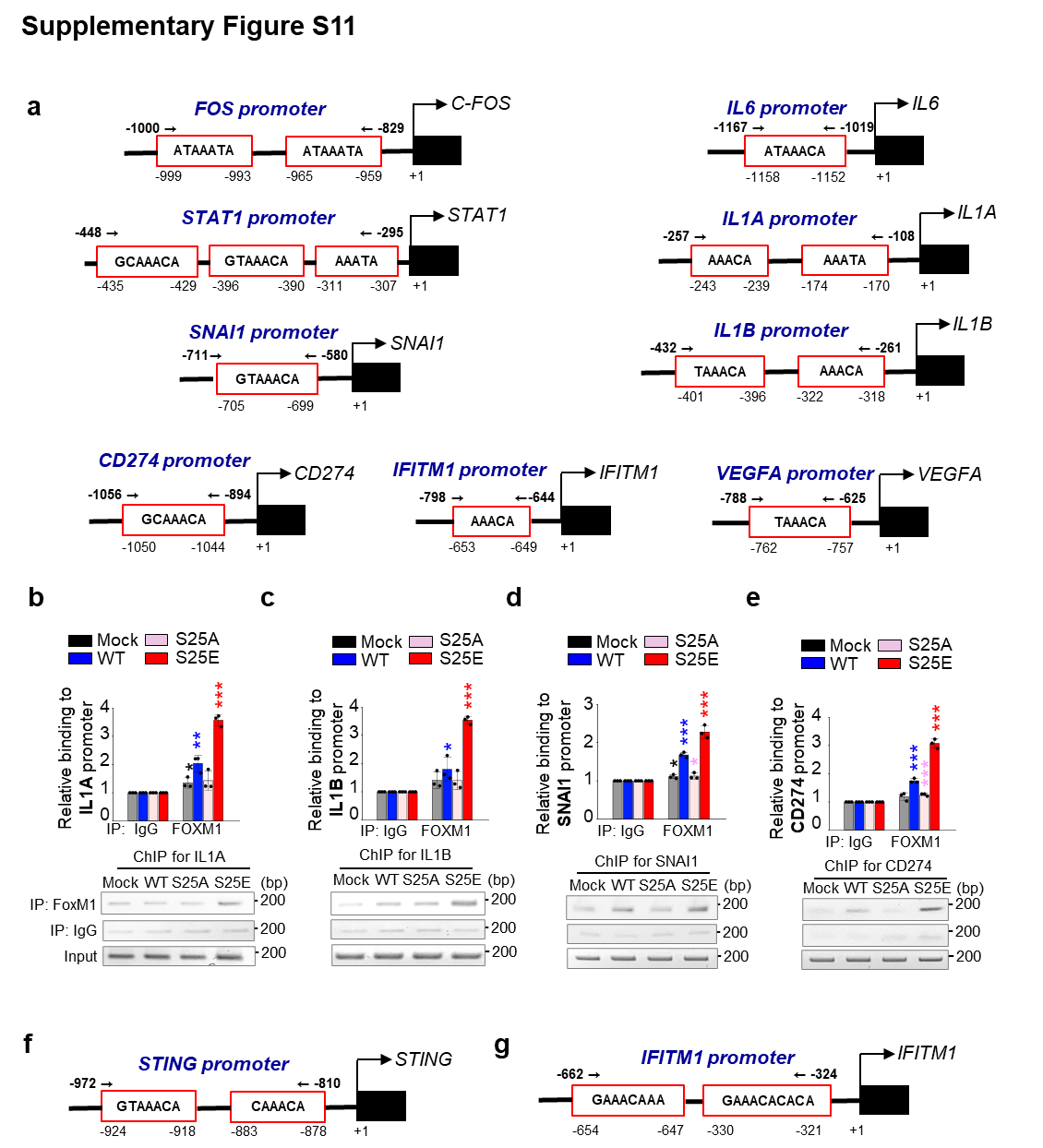
**

**Supplementary Figure S11.** **p-FoxM1^S25^ upregulates the genes related to immune escape, TAM polarization, and EMT by direct activation. a,** Scheme of promoter regions of *IFITM1*, *C-FOS*, *STAT1*, *CD274, IL6*, *IL1A*, *IL1B*, and *SNAI1*. FoxM1 binds to the region of A/G-C/T-AAA-C/T-A sequences^4^. **b-e,** ChIP assays for FoxM1 binding to promoters of *IL1A* (**b**), *IL1B* (**c**), *SNAI1* (**d**), or *CD274* (**e**). Assays were performed on chromatin fragments using antibody to FoxM1 and normalized to pre-immune normal IgG. Immunoprecipitated fractions were assayed by PCR for binding to the promoters of *IL1A* (**b**), *IL1B* (**c**), *SNAI1* (**d**), or *CD274* (**e**). The PCR products were visualized in agarose gel. **f,** Scheme of promoter regions of *STING* for binding FoxM1 to the region of A/G-C/T-AAA-C/T-A sequences^4^. **g,** Scheme of *IFITM1* promoter regions for binding IRF3 to the region of 5’-GAAA(G/C)(G/C)GAAA-3’ sequences^5^ of interferon-sensitive response element (ISRE).


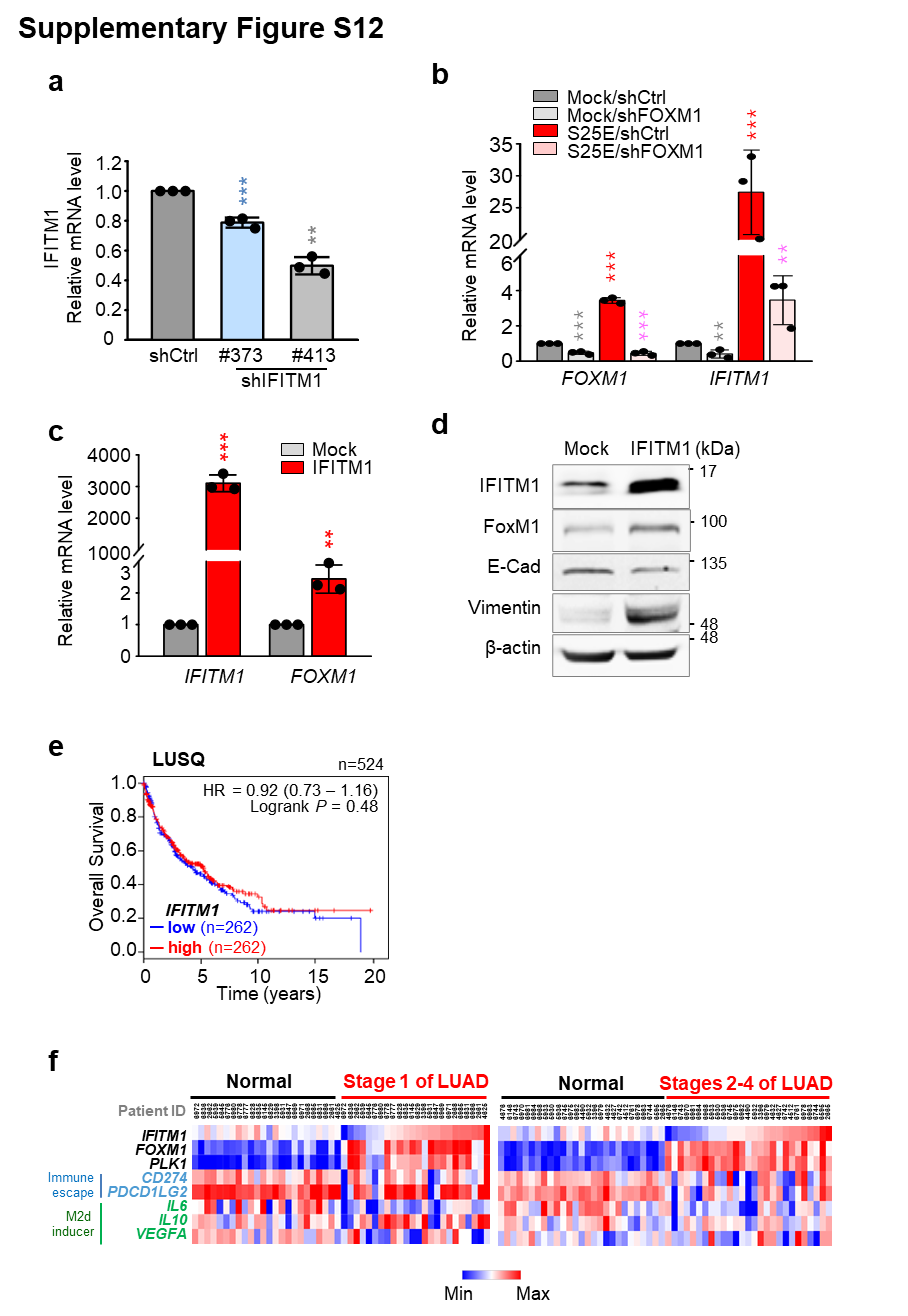


**Supplementary Figure S12.** **IFITM1 functions as a regulator of metastasis and polarization of M2d-TAM in p-FoxM1-induced metastasis. a,** IFITM1 shRNA targeting at the 373–393 or 413–433 position was treated with A549 cells. qRT-PCR was performed for *IFITM1.* ***p* < 0.01; ****p* < 0.001; (*n =* 3). **b,** FoxM1 shRNA was treated with A549 cells expressing S25E FoxM1 for 48 h. qRT-PCR was performed for *FOXM1* and *IFITM1.* ***p* < 0.01; ****p* < 0.001; (*n =* 3). **c-d,** IFITM1 plasmid was exogenously expressed in A549 cells for 40 h. The expression of FoxM1 and IFITM1 was observed using qRT-PCR (**c**) and immunoblot (**d**). **e,** Overall survival rates of patients in LUSQ were analyzed according to *IFITM1* expression level using KM PLOTTER. **f,** A heat map analysis showed the expression of genes *IFITM1*, *FOXM1*, *PLK1*, *CD274*, *PDCD1LG2*, *IL6*, *IL10*, and *VEGFA* based on the degree of expression of *IFITM1* from TCGA data analysis depending on stage of LUAD in normal and tumor tissue.

**Supplementary Tables**

**Supplementary Table S1.** List of primary antibodies used for immunoblot analysis.

| Antibody | Source | Cat. No. | Host Species | Working Concentration |
| --- | --- | --- | --- | --- |
| FoxM1 | Santa Cruz Biotechnology | sc500 | Rabbit | 1：1000 |
| PLK1 | Millipore | 05-844 | Mouse | 1：1000 |
| p-PLK1-T210 | Cell Signaling Technology | 5472S | Rabbit | 1：1000 |
| vimentin | Santa Cruz Biotechnology | sc7557 | Mouse | 1：1000 |
| E-cadherin | Cell Signaling | 4065 | Rabbit | 1：1000 |
| N-cadherin | Sigma-Aldrich | C3865 | Mouse | 1：1000 |
| SNAI1 | Santa Cruz Biotechnology | sc271977 | Mouse | 1：500 |
| SNAI2 | Cell Signaling Technology | 9585 | Rabbit | 1：1000 |
| p-Serine | Santa Cruz Biotechnology | sc81514 | Mouse | 1：1000 |
| p-Smad2 ^S465/S467^ | Cell Signaling Technology | 18338 | Rabbit | 1：1000 |
| Smad2/3 | Cell Signaling Technology | 8685 | Mouse | 1：1000 |
| GAPDH | Sigma-Aldrich | G8795 | Mouse | 1：10000 |
| β-actin | Sigma-Aldrich | A5441 | Mouse | 1：10000 |
| TCTP | Santa Cruz Biotechnology | sc133131 | Mouse | 1：1000 |
| p-TCTP | Cell Signaling Technology | 5251s | Rabbit | 1：1000 |
| RFP | Life Technologies | R10367 | Rabbit | 1：2000 |
| PD-L1 | Cell Signaling Technology | 13684 | Rabbit | 1：1000 |
| p-Erk1/2 | Cell Signaling Technology | 4370p | Rabbit | 1：1000 |
| Erk1/2 | Cell Signaling Technology | 4695p | Mouse | 1：1000 |
| c-Fos | Santa Cruz Biotechnology | sc52 | Mouse | 1：1000 |
| c-Jun | Santa Cruz Biotechnology | sc74543 | Mouse | 1：1000 |
| IFITM1 | ProteinTech Group | 60074 | Mouse | 1：1000 |
| IL1A | Santa Cruz Biotechnology | sc12741 | Mouse | 1：1000 |
| IL6 | Santa Cruz Biotechnology | sc28343 | Mouse | 1：1000 |
| STAT1 | Santa Cruz Biotechnology | sc464 | Mouse | 1：1000 |
| NF-κB | Santa Cruz Biotechnology | sc71677 | Mouse | 1：1000 |
| Histone H1 | Santa Cruz Biotechnology | sc8030 | Mouse | 1：1000 |
| STING | Santa Cruz Biotechnology | sc518172 | Mouse | 1：1000 |
| TBK1 | Santa Cruz Biotechnology | sc52957 | Mouse | 1：1000 |
| p-TBK1 | Cell Signaling Technology | 5483t | Rabbit | 1：1000 |
| IRF3 | Santa Cruz Biotechnology | sc33641 | Mouse | 1：1000 |
| CD68 | Santa Cruz Biotechnology | sc20060 | Mouse | 1：1000 |
| CD163 | ABclonal Science | A8383 | Rabbit | 1：1000 |

**Supplementary Table S2**. Sequences of forward (F) and reverse (R) primers used for qRT-PCR amplification.

| Target Gene | Primer | Sequences |
| --- | --- | --- |
| Human *FOXM1* | Forward  Reverse | 5’-TGGACCAGGTGTTTAAGCAGC-3’  5’-CCGGGAGACGTCTGCCAATGG-3’ |
| Human *PLK1* | Forward  Reverse | 5’- AAGAGATCCCGGAGGTCCTA -3’  5’- TCATTCAGGAAAAGGTTGCC -3’ |
| Human *CDH1* | Forward  Reverse | 5’- ACCACCTCCACAGCCACC -3’  5’- GTCCAGTTGGCACTCGCC -3’ |
| Human *CDH2* | Forward  Reverse | 5’- ACAGTGGCCACCTACAAAGG -3’  5’- CCGAGATGGGGTTGATAATG -3’ |
| Human *VIM* | Forward  Reverse | 5’- GAGAACTTTGCCGTTGAAGC -3’  5’- GCTTCCTGTAGGTGGCAATC -3’ |
| Human *SNAI1* | Forward  Reverse | 5’- GGAAGCCTAACTACAGCGAG -3’  5’- CAGAGTCCCAGATGAGCATTG -3’ |
| Human *SNAI2* | Forward  Reverse | 5’- ACGCCCAGCTACCCAATG -3’  5’- AGGGCGCCCAGGCTCACATA -3’ |
| Human *ZEB1* | Forward  Reverse | 5’-TGGGATCAACCACCAATGG-3’  5’-AAGTAACCCTGTGTATTTCTGGATGA-3’ |
| Human *TWIST* | Forward  Reverse | 5’- GGACAAGCTGAGCAAGATTCAGA-3’  5’- TCTGGAGGACCTGGTAGAGGAA -3’ |
| Human *IFITM1* | Forward  Reverse | 5’-TCAACACCCTCTTCTTGAAC-3’  5’-AGAGCCGAATACCAGTAACA-3’ |
| Human *XAF1* | Forward  Reverse | 5’-TTCGTCCAAAGACAAGAAGT-3’  5’-CTCCTCAGAATGTCATAGGC-3’ |
| Human *IL1A* | Forward  Reverse | 5’-AAGTCATCAAAGGATGATGC-3’  5’-AGAAGAAGAGGAGGTTGGTC-3’ |
| Human *IL1B* | Forward  Reverse | 5’-GGGCCTCAAGGAAAAGAATC-3’  5’-TTCTGCTTGAGAGGTGCTGA-3’ |
| Human *MX1* | Forward  Reverse | 5’-CAAAGGAACTGAAGACAAGG-3’  5’-GAAATATGGGTGGTTCTCAA-3’ |
| Human *IFI44L* | Forward  Reverse | 5’-GTCTTGAAGAACCTCACTGC-3’  5’-GGATTCACTGTGTGGCTTAT-3’ |
| Human *INOS* | Forward  Reverse | 5’-TATCACAACCTCAGCAAGCA-3’  5’-AAAATCCCTTTGGCCTTATG-3’ |
| Human *IL12B* | Forward  Reverse | 5’-GGAGCTGCTACACTCTCTGC-3’  5’-GATGAAGAAGCTGCTGGTGT-3’ |
| Human *IL4* | Forward  Reverse | 5’-ACATTGTCACTGCAAATCGACACC-3’  5’-TGTCTGTTACGGTCAACTCGGTGC-3’ |
| Human *IL6* | Forward  Reverse | 5’-CAGACAGCCACTCACCTCTT-3’  5’-CTTTTTCAGCCATCTTTGGA-3’ |
| Human *IL10* | Forward  Reverse | 5’-AACCAAGACCCAGACATCAA-3’  5’-TGGCTTTGTAGATGCCTTTC -3’ |
| Human *CD163* | Forward  Reverse | 5’-TGATTCGGACTTCTCTCTGG-3’  5’-TGGCTACAAGTTCCTTCTGG-3’ |
| Human *CD206* | Forward  Reverse | 5’-ACTGCAAGCTTCACAATTCC-3’  5’-ATTTCAATTTGGGCTCATCA-3’ |
| Human *CD274* | Forward  Reverse | 5’-CAAAGAATTTTGGTTGTGGA-3’  5’-AGCTTCTCCTCTCTCTTGGA-3’ |
| Human *TGFB1* | Forward  Reverse | 5’-GGGACTATCCACCTGCAAGA-3’  5’-CCTCCTTGGCGTAGTAGTCG-3’ |
| Human *VEGFA* | Forward  Reverse | 5’-TTCCAGGAGTACCCTGATGA-3’  5’-TGAGGTTTGATCCGCATAAT-3’ |
| Human *CD279* | Forward  Reverse | 5’-CGTGGCCTATCCACTCCTCA-3’  5’-ATCCCTTGTCCCAGCCACTC-3’ |
| Human *CXCL1* | Forward  Reverse | 5’-ACCCCAAGAACATCCAAAGT -3  ’5’-TGGATTTGTCACTGTTCAGC -3’ |
| Human *GAPDH* | Forward  Reverse | 5’- TAAAGGGCATCCTGGGCTACACT -3’  5’- TTACTCCTTGGAGGCCATGTAGG -3’ |
| Mouse *INOS* | Forward  Reverse | 5’-CCTGTGTTCCACCAGGAGAT-3’  5’-AGAGGACTGTGGCTCTGACC-3’ |
| Mouse *IL12B* | Forward  Reverse | 5’-CTGCAGAGAAGGTCACACTG-3’  5’-TGATGATGTCCCTGATGAAG-3’ |
| Mouse *IL10* | Forward  Reverse | 5’-CAGCCGGGAAGACAATAACT-3’  5’-TCATTTCCGATAAGGCTTGG-3’ |
| Mouse *CD163* | Forward  Reverse | 5’-AGATTGCCTCATGACTGCTC-3’  5’-CACTTGCTATGCAGGGAACT-3’ |
| Mouse *CD206* | Forward  Reverse | 5’-GAGCCTGTGAGCAACCACTA-3’  5’-TTTCATTTGTGCATGTGTGG-3’ |
| Mouse *VEGFA* | Forward  Reverse | 5’-TTAAATCCTGGAGCGTTC-3’  5’-CACATCTGCAAGTACGTTCG-3’ |
| Mouse *GAPDH* | Forward  Reverse | 5’-GTTGTCTCCTGCGACTTCA-3’  5’-GGTGGTCCAGGGTTTCTTA-3’ |

**Supplementary Table S3.** Sequences of forward (F) and reverse (R) primers used for site-directed mutagenesis for FoxM1.

| Target Residue | Primer | Sequences |
| --- | --- | --- |
| S25A | Forward  Reverse | 5’-CCTCCTCTGATGTTTCAGCTGGGGCATTTTGAACAGGAAG -3’  5’-CTTCTTGTTCAAAATGCCCCAGCTGAAACATCAGAGGAGG -3’ |
| S25E | Forward  Reverse | 5’-CTTCCTGTTCAAAATGCCCCAGAGGAAACATCAGAGGAGGAACCT -3’  5’-AGGTTCCTCCTCTGATGTTTCCTCTGGGGCATTTTGAACAGGAAG -3’ |
| S361A | Forward  Reverse | 5’-TAGGTACCAGGTATGCGCTGACCCGTGGTAG -3’  5’-CTACCACGGGTCAGCGCATACCTGGTACCTA -3’ |
| S361E | Forward  Reverse | 5’-CTGCTACCACGGGTCAGCGAGTACCTGGTACCTATCCAG-3’  5’-CTGGATAGGTACCAGGTACTCGCTGACCCGTGGTAGCAG -3’ |
| S715A | Forward  Reverse | 5’-AGCAGGATCTTGCTGAGGGCGTCATTCATTGTGTCCAG -3’  5’-CTGGACACAATGAATGACGCCCTCAGCAAGATCCTGCT -3’ |
| S715E | Forward  Reverse | 5’-CCTGGACACAATGAATGACGAGCTCAGCAAGATCCTGCTGG -3’  5’-CCAGCAGGATCTTGCTGAGCTCGTCATTCATTGTGTCCAGG -3’ |

**Supplementary Table S4.** Sequences of forward (F) and reverse (R) primers used for ChIP assay.

| Target Gene | Primer | Sequences |
| --- | --- | --- |
| Human *IL6* | Forward  Reverse | 5’-TATAGGTGAATAAACAAG -3’  5’-CCTTTTCCCAGAGGTAGCAC -3’ |
| Human *VEGFA* | Forward  Reverse | 5’-CGGGTTTTATCCCTCTTCTT -3’  5’-TTCTGCTGGTTTCCAAAATC -3’ |
| Human *STAT1* | Forward  Reverse | 5’-AGTTTGGGCTTCTGCAAACA -3’  5’-CACGCGCTGGGGTATTTC -3’ |
| Human *FOS* | Forward  Reverse | 5’-AATAAATGCGCTGTCTTCTTT -3’  5’-CAGACCTTCATCCCCTAACC -3’ |
| Human *IL1A* | Forward  Reverse | 5’-CTGGAATATCTGCAAACAAC -3’  5’-TGGCTACGTGGCTACAAGTG -3’ |
| Human *IL1B* | Forward  Reverse | 5’-CTGGAATATCTGCAAACAAC -3’  5’-TGGCTACGTGGCTACAAGTG -3’ |
| Human *IFITM1* | Forward  Reverse | 5’-GGTTTTATTGAGCAGAGTGA -3’  5’-GCCTTTGTTTCTCTCTCTGG -3’ |
| Human *SNAI1* | Forward  Reverse | 5’-CGCTCCGTAAACACTGGATA -3’  5’-AGGGAAACGCACATCACTG -3’ |
| Human *CD274* | Forward  Reverse | 5’-GGAAAGGCAAACAACGAAGA -3’  5’-AGGGAAACGCACATCACTG -3’ |

**Supplementary Table S5.** Analysis of Spearman’s and Pearson’s coefficients for the correlations between cell cycle-regulatory factors *PLK1*, *FOXM1*, *CCNA1*, *CCNB1*, *CCND1*, *CCNE1*, *CDK1*, *CDK2*, *PCNA*, and *MKI67* in non-small cell lung cancer (NSCLC) patients using cBioPortal used in Figure 1.

| Genes | Spearman | | Pearson | |
| --- | --- | --- | --- | --- |
|  | Coefficient | *P* value | Coefficient | *P* value |
| *PLK1 & FOXM1* | 0.88 | 1.68E-55 | 0.86 | 3.22E-51 |
| *PLK1 & CCNA1* | -0.01 | 0.896 | 0.02 | 0.749 |
| *PLK1 & CCNB1* | 0.81 | 7.40E-41 | 0.77 | 5.37E-35 |
| *PLK1 & CCND1* | -0.24 | 1.54E-03 | -0.3 | 8.77E-05 |
| *PLK1 & CCNE1* | 0.79 | 5.03E-37 | 0.75 | 7.67E-32 |
| *PLK1 & CDK1* | 0.68 | 1.09E-24 | 0.67 | 2.37E-23 |
| *PLK1 & CDK2* | 0.46 | 4.84E-10 | 0.44 | 1.44E-09 |
| *PLK1 & PCNA* | 0.53 | 2.07E-13 | 0.54 | 3.56E-14 |
| *PLK1 & MKI67* | 0.89 | 3.67E-59 | 0.89 | 1.13E-59 |
| *FOXM1 & CCNA1* | -0.06 | 0.419 | -0.01 | 0.858 |
| *FOXM1 & CCNB1* | 0.81 | 2.67E-41 | 0.79 | 6.43E-38 |
| *FOXM1 & CCND1* | -0.15 | 0.0481 | -0.23 | 2.84E-03 |
| *FOXM1 & CCNE1* | 0.84 | 3.51E-47 | 0.83 | 1.73E-44 |
| *FOXM1 & CDK1* | 0.71 | 2.25E-27 | 0.73 | 1.19E-29 |
| *FOXM1 & CDK2* | 0.43 | 4.71E-09 | 0.4 | 5.20E-08 |
| *FOXM1 & PCNA* | 0.48 | 2.41E-11 | 0.5 | 2.96E-12 |
| *FOXM1 & MKI67* | 0.87 | 1.87E-53 | 0.85 | 5.72E-48 |
| *CCNA1 & CCNB1* | 0.01 | 0.906 | 0.05 | 0.542 |
| *CCNA1 & CCND1* | -0.1 | 0.191 | -0.09 | 0.256 |
| *CCNA1 & CCNE1* | 0 | 0.985 | 0.04 | 0.643 |
| *CCNA1 & CDK1* | -0.02 | 0.748 | 0.01 | 0.928 |
| *CCNA1 & CDK2* | -0.11 | 0.165 | -0.07 | 0.372 |
| *CCNA1 & PCNA* | -0.07 | 0.346 | -0.06 | 0.453 |
| *CCNA1 & MKI67* | -0.05 | 0.522 | 0 | 0.958 |
| *CCNB1 & CCND1* | -0.23 | 2.90E-03 | -0.26 | 5.04E-04 |
| *CCNB1 & CCNE1* | 0.78 | 7.44E-36 | 0.77 | 2.09E-34 |
| *CCNB1 & CDK1* | 0.82 | 4.23E-42 | 0.82 | 7.23E-42 |
| *CCNB1 & CDK2* | 0.41 | 3.16E-08 | 0.37 | 6.08E-07 |
| *CCNB1 & PCNA* | 0.66 | 3.33E-22 | 0.61 | 2.15E-18 |
| *CCNB1 & MKI67* | 0.74 | 3.86E-31 | 0.7 | 1.05E-26 |
| *CCND1 & CCNE1* | -0.33 | 1.45E-05 | -0.45 | 1.07E-09 |
| *CCND1 & CDK1* | -0.19 | 0.0117 | -0.28 | 2.57E-04 |
| *CCND1 & CDK2* | -0.14 | 0.0698 | -0.3 | 6.80E-05 |
| *CCND1 & PCNA* | -0.19 | 0.0143 | -0.34 | 7.34E-06 |
| *CCND1 & MKI67* | -0.15 | 0.0501 | -0.19 | 0.0141 |
| *CCNE1 & CDK1* | 0.72 | 5.84E-28 | 0.72 | 2.58E-28 |
| *CCNE1 & CDK2* | 0.4 | 9.94E-08 | 0.41 | 3.56E-08 |
| *CCNE1 & PCNA* | 0.57 | 1.12E-15 | 0.57 | 3.97E-16 |
| *CCNE1 & MKI67* | 0.78 | 4.07E-35 | 0.73 | 3.11E-29 |
| *CDK1 & CDK2* | 0.5 | 3.54E-12 | 0.52 | 5.57E-13 |
| *CDK1 & PCNA* | 0.64 | 7.39E-21 | 0.63 | 3.06E-20 |
| *CDK1 & MKI67* | 0.63 | 8.86E-20 | 0.6 | 6.38E-18 |
| *CDK2 & PCNA* | 0.37 | 7.07E-07 | 0.43 | 3.84E-09 |
| *CDK2 & MKI67* | 0.43 | 5.09E-09 | 0.41 | 2.35E-08 |
| *PCNA & MKI67* | 0.44 | 2.44E-09 | 0.43 | 7.05E-09 |

**Supplementary Table S6.** The specific number of patients with NSCLC and LUAD for KM plots in Figures 1 and 8 (*N/A: Not applicable).

|  | Number of patients | | | Number of LUAD patients | | | | | |
| --- | --- | --- | --- | --- | --- | --- | --- | --- | --- |
| No. of Dataset | NSCLC | LUAD | LUSQ | Sex  (F/M) | Stage 1 | Stage 2 | Stage 3 | Stage 4 | *N/A |
| GSE68465 | 442 | - | - | 220/222 | - | - | - | - | 442 |
| GSE14814 | 123 | 71 | 52 | 34/37 | 42 | 29 | - | - | - |
| GSE19188 | 82 | 40 | 24 | 15/25 | - | - | - | - | 40 |
| GSE29013 | 55 | 30 | 25 | 10/20 | 16 | 6 | 8 | - | - |
| GSE30219 | 293 | - | 82 | - | - | - | - | - | - |
| GSE31210 | 226 | 226 | - | 121/105 | 168 | 58 | - | - | - |
| GSE3141 | 111 | 58 | 53 | - | - | - | - | - | 58 |
| GSE31908 | 46 | 45 | - | 34/11 | 17 | 8 | 5 | - | 15 |
| GSE37745 | 196 | 106 | 66 | 60/46 | 70 | 19 | 13 | 4 | - |
| GSE4573 | 130 | - | 130 | - | - | - | - | - | - |
| GSE50081 | 181 | 127 | 42 | 62/65 | 92 | 35 | - | - | - |
| TCGA | - | - | - | 277/238 | 275 | 122 | 81 | 26 | 8 |

**Supplementary Table S7.** Cox regression analysis for survival of non-small cell lung cancer (NSCLC) and lung adenocarcinoma (LUAD) patients expressing PLK1 and FOXM1 of KM plot used in Figure 1.

| Patients | Endpoint | Gene expression | Number of patients (n) | Hazard ratio (HR) | 95% Confidential interval (CI) |
| --- | --- | --- | --- | --- | --- |
| NSCLC | Overall Survival (OS)  n=1885 | FOXM1^Hi^/PLK1^Hi^ | 732 | 1.602 | 1.3822 ~ 1.857 |
|  |  | FOXM1^Hi^/PLK1^Lo^ | 212 | 1.673 | 1.3581 ~ 2.062 |
|  |  | FOXM1^Lo^/PLK1^Hi^ | 213 | 1.131 | 0.9022 ~ 1.417 |
|  |  | FOXM1^Lo^/PLK1^Lo^ | 728 | - | - |
| LUAD | Overall Survival (OS)  n=703 | FOXM1^Hi^/PLK1^Hi^ | 260 | 2.172 | 1.6314 ~ 2.891 |
|  |  | FOXM1^Hi^/PLK1^Lo^ | 93 | 1.455 | 0.9862 ~ 2.147 |
|  |  | FOXM1^Lo^/PLK1^Hi^ | 93 | 1.030 | 0.6727 ~ 1.578 |
|  |  | FOXM1^Lo^/PLK1^Lo^ | 257 | - | - |
| Stages 3-4 LUAD | Overall Survival (OS)  n=137 | FOXM1^Hi^/PLK1^Hi^ | 65 | 1.904 | 1.1501 ~ 2.550 |
|  |  | FOXM1^Hi^/PLK1^Lo^ | 13 | 0.804 | 0.3439 ~ 1.880 |
|  |  | FOXM1^Lo^/PLK1^Hi^ | 8 | 0.764 | 0.2289 ~ 2.550 |
|  |  | FOXM1^Lo^/PLK1^Lo^ | 51 | - | - |

**Supplementary Table S8.** Clinical information about LUAD patients including their differentiation stages for an immunohistochemistry analysis in Figure 1e.

| Sample | Age | Gender | Cat. No. | Histologic Classifications | Tumor grade |
| --- | --- | --- | --- | --- | --- |
| Normal | 62 | Male | 0028000B | Lung Normal | - |
| LUAD #1 | 62 | Male | 2881403A | Lung Adenocarcinoma,  Well differentiated (WD) | Grade 1,  Low-grade |
| LUAD #2 | 62 | Male | 2881403B | Lung Adenocarcinoma,  Moderately differentiated (MD) | Grade 2,  Intermediate- grade |
| LUAD # 3 | 63 | Male | 2881403C | Lung Adenocarcinoma,  Poorly differentiated (PD) | Grade 3,  High-grade |

**Supplementary Table S9.** Cox regression analysis for overall survival of lung adenocarcinoma (LUAD) patients expressing *PLK1*, *FOXM1*, and *IFITM1* of KM plot used in Figure 8i.

| Tumor Stage | Gene expression | Number of patients (n) | Hazard ratio (HR) | 95% Confidential interval (CI) |
| --- | --- | --- | --- | --- |
| All  (n=703) | FOXM1^Hi^/PLK1^Hi^/ IFITM ^Hi^ | 156 | 2.236 | 1.5299 ~ 3.267 |
|  | FOXM1^Hi^/PLK1^Hi^/ IFITM ^Lo^ | 104 | 2.337 | 1.5658 ~ 3.488 |
|  | FOXM1^Hi^/PLK1^Lo^/ IFITM ^Hi^ | 49 | 1.599 | 0.9539 ~ 2.680 |
|  | FOXM1^Hi^/PLK1^Lo^/ IFITM ^Lo^ | 44 | 1.440 | 0.8181 ~ 2.536 |
|  | FOXM1^Lo^/ PLK1^Hi^/ IFITM ^Hi^ | 40 | 1.224 | 0.6675 ~ 2.244 |
|  | FOXM1^Lo^/ PLK1^Hi^/ IFITM ^Lo^ | 53 | 0.980 | 0.5498 ~ 1.748 |
|  | FOXM1^Lo^/ PLK1^Lo^/IFITM1^Hi^ | 109 | 1.121 | 0.7075 ~ 1.776 |
|  | FOXM1^Lo^/ PLK1^Lo^/IFITM1^Lo^ | 148 | - | - |
| Stages  3-4  (n=137) | FOXM1^Hi^/PLK1^Hi^/ IFITM ^Hi^ | 32 | 2.439 | 1.2065 ~ 4.931 |
|  | FOXM1^Hi^/PLK1^Hi^/ IFITM ^Lo^ | 33 | 1.751 | 0.8420 ~ 3.643 |
|  | FOXM1^Hi^/PLK1^Lo^/ IFITM ^Hi^ | 6 | 1.597 | 0.5545 ~ 4.599 |
|  | FOXM1^Hi^/PLK1^Lo^/ IFITM ^Lo^ | 7 | 0.400 | 0.0894 ~ 1.788 |
|  | FOXM1^Lo^/PLK1^Hi^/ IFITM ^Hi^ | 4 | 3.335 | 0.7223 ~ 15.384 |
|  | FOXM1^Lo^/PLK1^Hi^/ IFITM ^Lo^ | 4 | 0.318 | 0.0416 ~ 2.439 |
|  | FOXM1^Lo^/ PLK1^Lo^/ IFITM ^Hi^ | 28 | 1.174 | 0.5057 ~ 2.725 |
|  | FOXM1^Lo^/ PLK1^Lo^/ IFITM ^Lo^ | 23 | - | - |

**Supplementary Table S6.** Sequences of forward (F) and reverse (R) primers used for ChIP assay.

| Target Gene | Primer | Sequences |
| --- | --- | --- |
| Human *IL6* | Forward  Reverse | 5’-TATAGGTGAATAAACAAG -3’  5’-CCTTTTCCCAGAGGTAGCAC -3’ |
| Human *VEGFA* | Forward  Reverse | 5’-CGGGTTTTATCCCTCTTCTT -3’  5’-TTCTGCTGGTTTCCAAAATC -3’ |
| Human *STAT1* | Forward  Reverse | 5’-AGTTTGGGCTTCTGCAAACA -3’  5’-CACGCGCTGGGGTATTTC -3’ |
| Human *FOS* | Forward  Reverse | 5’-AATAAATGCGCTGTCTTCTTT -3’  5’-CAGACCTTCATCCCCTAACC -3’ |
| Human *IL1A* | Forward  Reverse | 5’-CTGGAATATCTGCAAACAAC -3’  5’-TGGCTACGTGGCTACAAGTG -3’ |
| Human *IL1B* | Forward  Reverse | 5’-CTGGAATATCTGCAAACAAC -3’  5’-TGGCTACGTGGCTACAAGTG -3’ |
| Human *IFITM1* | Forward  Reverse | 5’-GGTTTTATTGAGCAGAGTGA -3’  5’-GCCTTTGTTTCTCTCTCTGG -3’ |
| Human *SNAI1* | Forward  Reverse | 5’-CGCTCCGTAAACACTGGATA -3’  5’-AGGGAAACGCACATCACTG -3’ |
| Human *CD274* | Forward  Reverse | 5’-GGAAAGGCAAACAACGAAGA -3’  5’-AGGGAAACGCACATCACTG -3’ |

**Reference**

1. Shin, S.B., Jang, H.R., Xu, R., Won, J.Y. & Yim, H. Active PLK1-driven metastasis is amplified by TGF-beta signaling that forms a positive feedback loop in non-small cell lung cancer. *Oncogene* **39**, 767-785 (2020).

2. Gyorffy, B., Surowiak, P., Budczies, J. & Lanczky, A. Online survival analysis software to assess the prognostic value of biomarkers using transcriptomic data in non-small-cell lung cancer. *PLoS One* **8**, e82241 (2013).

3. Clarke, D.J.B. *et al.* Appyters: Turning Jupyter Notebooks into data-driven web apps. *Patterns (N Y)* **2**, 100213 (2021).

4. Chen, X. *et al.* Targeting the CtBP1-FOXM1 transcriptional complex with small molecules to overcome MDR1-mediated chemoresistance in osteosarcoma cancer stem cells. *J Cancer* **12**, 482-497 (2021).

5. Andrilenas, K.K. *et al.* DNA-binding landscape of IRF3, IRF5 and IRF7 dimers: implications for dimer-specific gene regulation. *Nucleic Acids Res* **46**, 2509-2520 (2018).
